# Characterising the Exposure of Prison Staff to Second-Hand Tobacco Smoke

**DOI:** 10.1093/annweh/wxx058

**Published:** 2017-07-16

**Authors:** Sean Semple, Helen Sweeting, Evangelia Demou, Greig Logan, Rachel O’Donnell, Kate Hunt, on behalf of the Tobacco in Prisons (TIPs) Research Team

**Affiliations:** 1 Respiratory Group, Division of Applied Health Sciences, University of Aberdeen, Aberdeen AB25 2ZG, UK;; 2 MRC/CSO Social and Public Health Sciences Unit, Institute of Health and Wellbeing, University of Glasgow, 200 Renfield Street, Glasgow G2 3QB, UK

**Keywords:** correctional facilities, ETS, nicotine, PM_2.5_, SHS, work

## Abstract

Second-hand tobacco smoke (SHS) is an avoidable and harmful exposure in the workplace but >25000 prison staff continue to be exposed on a daily basis in the UK and many more worldwide. SHS exposures in prisons are incompletely understood but may be considerable given the large proportion of smoking prisoners and limited ventilation. This study characterized the exposure of prison staff to SHS in all 15 prisons in Scotland using multiple methods. Exposure assessment strategies included 6-day area measurement of fine Particulate Matter (PM_2.5_) and airborne nicotine in each prison together with short (30-minute) measurements of PM_2.5_ covering a range of locations/activities. Pre- and post-shift saliva samples were also gathered from non-smoking staff and analysed for cotinine to estimate exposure. There was evidence of exposure to SHS in all prisons from the results of PM_2.5_ and nicotine measurements. The salivary cotinine results from a sub-sample of non-smoking workers indicated SHS exposures of similar magnitude to those provided by the 6-day area measurements of PM_2.5_. There was a high degree of exposure variability with some locations/activities involving exposure to SHS concentrations that were comparable to those measured in bars in Scotland prior to smoke-free legislation in 2006. The median shift exposure to SHS-PM_2.5_ was ~20 to 30 µg m^−3^ and is broadly similar to that experienced by someone living in a typical smoking home in Scotland. This is the most comprehensive assessment of prison workers’ exposure to SHS in the world. The results are highly relevant to the development of smoke-free policies in prisons and should be considered when deciding on the best approach to provide prison staff with a safe and healthy working environment.

## Introduction

Exposure to second-hand tobacco smoke (SHS) has been known to be harmful to health for at least 35 years (Garfinkel, 1981). Restrictions on smoking in enclosed public spaces, including most workplaces, were implemented in 2006 in Scotland and 2007 elsewhere in the UK. Such smoke-free laws decrease workers’ SHS exposure (Semple *et al.*, 2007a) with direct health benefit to workers (Ayres *et al.*, 2009) and at a wider population level (Pell *et al.*, 2008).

In the UK, prisons—as both workplaces for staff and prisoners’ ‘homes’—have been exempt from smoke-free legislation. They are one of the few institutions in which smoking remains normative: recent data for Scotland indicate that nearly three-quarters (72%) of prisoners smoke (Scottish Prison Service, 2015).

There is international interest in finding suitable methods for greater tobacco control in prison to benefit prisoner/staff health and combat inequalities (Butler *et al.*, 2007; Baybutt *et al.*, 2014). In September 2015, a phased roll-out of smoke-free policies in four Welsh prisons and four pilot prisons in England was announced. Smoking restrictions in prisons have also been introduced in the USA, Switzerland, New Zealand, and Australia. In Scotland, prisoners are permitted to smoke: within cells accommodating single individuals, within cells accommodating two or more individuals unless these have been designated as non-smoking, and during outdoor recreation (restricted to certain outdoor areas in some prisons). Staff and visitors are not allowed to smoke anywhere within prison boundaries. The Scottish Government sees a smoke-free prison service as a key step towards a smoke-free Scotland (Scottish Government, 2013) and is committed to finalising plans that set out how indoor smoke-free prison facilities will be delivered. Part of the momentum for this has come from the need to protect prison workers’ health. Staff within Scottish prisons work in a wide variety of roles, including residential officers (working in the cell and hallway/landings areas), operational officers (patrolling, escorting prisoners, prisoner reception, visits, ‘front of house’), those working to train prisoners in vocational skills or physical education, management and support roles (e.g. finance, psychology), and engineering (designing, installing, and maintaining systems across the prison estate). Some of these roles require staff to enter areas where smoking occurs.

There are two particular problems in characterising occupational exposure to SHS. The first is that there is no UK Workplace Exposure Limit or international equivalent, and the second is that there is no standard method for assessing workplace SHS exposure. Previous work in the hospitality industry has used the concentration of fine particulate matter (PM_2.5_; Semple *et al.*, 2007b) or airborne concentrations of nicotine (Mulcahy *et al.*, 2005) to provide data on the effect of implementing smoke-free regulations or policy changes. Some studies have also used measures of salivary cotinine, a biomarker of exposure to nicotine (Lawhorn *et al.*, 2013). Research studies on occupational exposure to SHS have therefore tended to utilize environmental exposure guidance values from the World Health Organisation (WHO, 2010) as benchmarks to provide some indication of the potential harm from SHS concentrations measured as PM_2.5_. Comparison with these values should be considered alongside the WHO’s scientific consensus statement that ‘there is no safe level of exposure to SHS’ (WHO, 2010).

Recent studies which have examined prison staff exposure to SHS have reported on area or fixed location PM_2.5_ (or PM_10_) and/or nicotine concentrations measured over varying periods in one to six prisons (Hammond & Emmons, 2005; Proescholdbell *et al.*, 2008; Ritter *et al.*, 2012; Thornley *et al.*, 2013; Semple *et al.*, 2015a; Jayes *et al.*, 2016; He *et al.*, 2016).

The Tobacco In Prisons study (TIPs) is a three-phase evaluation of graduated progress towards smoke-free prisons in Scotland: Phase 1 has obtained baseline values for smoking, SHS exposure and relevant health indicators, and social norms around smoking in Scotland’s 15 prisons; Phase 2 will entail a process evaluation of initiatives in anticipation of increased restrictions on smoking in prisons; Phase 3 will evaluate the impact of the implementation of smoke-free policies on health, economic, cultural, and organisational outcomes and will only proceed if such policies are introduced in Scotland. TIPs offers the opportunity to characterize prison workers’ exposure to SHS across all prisons within a national jurisdiction and to provide the most globally comprehensive evaluation of changes in prison workers’ exposure to SHS that result from steps towards implementation of a national smoke-free prison policy (Hunt *et al.*, 2017).

This paper aims to characterize prison workers’ exposure to SHS prior to any such changes, as assessed by a suite of complementary methods, to consider differences in the concentrations of SHS experienced within prisons and across the prison service, examine the strengths and weaknesses of each methodology employed, and determine which methods are most suitable for re-deployment in a future evaluation/post-implementation phase.

## Methods

### Study overview

Four complementary methods were used to quantify the exposure of prison staff to SHS. These were:

- Area measurement of PM_2.5_ concentrations over a 6-day period within a hall or landing area in each prison
- Area measurement of airborne nicotine concentrations over a 6-day period within a hall or landing area in each prison
- ‘Mobile’ measurement of PM_2.5_ concentrations in specific locations or during particular activities
- Measurement of cotinine concentrations in the saliva of non-smoking staff at both the beginning and end of a work-shift

Full details of these methods are provided in the following sections.

The study was carried out in all 15 of the prisons in Scotland operated by, or on behalf of, the Scottish Prison Service. Staff from all of the 15 prison establishments in Scotland were involved in gathering airborne PM_2.5_ and nicotine data with support from the study team, following a half-day training session. The aim of this session was to provide these staff with the background to the TIPs research, to explain the methods that would be used to assess the exposure of prisons staff to SHS and to provide basic training in the use of two air quality measuring devices.

The protocol and study tools were reviewed and gained approval from the Scottish Prison Service Research Access and Ethics Committee and the University of Glasgow College of Social Sciences Ethics Committee for Non-Clinical Research Involving Human Subjects (ref number: 400150213).

### Area or fixed monitoring

SHS concentrations were estimated via measurement of (i) fine Particulate Matter (PM_2.5_) and (ii) airborne nicotine concentrations over a sustained period (up to 6 days) in a single location in each prison. At least one trained staff member from each prison took responsibility for the placement, checking, and retrieval of the instruments. A form recording the location of the device, on/off dates and times and contextual information was completed.

PM_2.5_ was measured using a Dylos DC1700 monitor to log airborne concentration of particles every minute (Semple *et al.*, 2013) while the nicotine concentration was measured using a sodium bisulfate treated filter in a passive diffusion monitor (Hammond and Leaderer, 1987) to provide an overall average concentration of nicotine over the total sampling period. Devices were placed together at a secure location where an electrical power outlet was available in an atrium or landing of one residential hall in each prison; exact locations were chosen by prison staff who made pragmatic decisions to allow 6 days continuous monitoring whilst protecting the devices from malicious or accidental interference. The Dylos and nicotine monitors were located within 1 m of each other to gather directly comparable, contemporaneous measurements. In two prisons, duplicate nicotine monitors were placed to determine the accuracy of the method. The nicotine monitor was unsealed to expose its membrane to the environment at the same time as the Dylos machine was switched on to commence the area reading. On completion of area monitoring, the Dylos machine was switched off and the nicotine monitor sealed and bagged following a standard protocol.

Study team staff downloaded the Dylos data using Dylos Logger software. The Dylos DC1700 measures and records the concentration of particles in two size ranges: >0.5 µm and >2.5 µm diameter. By subtracting the latter from the former, it is possible to estimate the number and hence the mass concentration of particles between 0.5 and 2.5 µm using previously published equations for exposure to SHS aerosol (Semple *et al.*, 2015b). Each Dylos device had a specific calibration factor applied from a chamber experiment where measured concentrations of SHS-PM_2.5_ were compared to those reported from a TSI Sidepak AM510 Personal Aerosol Monitor itself set to a correction factor of 0.295 for SHS aerosol (Jiang *et al.*, 2011).

After this 6-day measurement period, the nicotine monitors were retrieved by research staff and transported to Aberdeen, UK, before being sent by airfreight for analysis at John Hopkins School of Public Health, Baltimore, USA. Two field blanks were transported and stored in an identical fashion to the monitors used in the prisons but were not exposed to the air. The filters in each monitor were extracted with an internal standard (isoquinoline, Sigma-Aldrich, St. Louis, MO, USA) and analysed using a gas chromatograph with a nitrogen phosphorus detector (GC-FTD, Shimadzu GC-2014, Shimadzu, Columbia, MD, USA). Nicotine was separated using a capillary column (SHRXI-5MS, Shimadzu). The analytical limit of detection (LOD) based on a 6-day measurement period was 0.031 µg m^−3^ of nicotine. For the purposes of presenting summary statistics, values of ½ the LOD (0.016 µg m^−3^) were employed for filters below the LOD. Both field blanks were below the LOD indicating that no nicotine contamination was likely to have taken place during the transport or storage of the monitors.

### Mobile monitoring of PM_2.5_

After the area monitoring data were downloaded, the Dylos device was returned to the staff member responsible for air quality measurement. They then carried out a series of mobile and activity-based measurements across their prison. The timing and location of these measurements were at the discretion of the staff member, to reflect operational requirements and local concerns about SHS exposure. They were asked to provide between four and eight 30-minute measurements in a range of location types such as cells, offices, reception areas, workshops, on-landing activity, and during any duties where they suspected SHS exposure may occur. The Dylos’ DC1700 internal battery enables it to be carried by prison staff shadowing workers performing duties such as cell unlocking, cell searches, etc. A form, describing the time and location of the measurement and the associated activity being undertaken, was completed. On completion of these mobile measurements, the Dylos DC1700 was collected and the data downloaded and analysed as described for the area monitoring. The results from the mobile measurements were pooled from all 15 prisons and used to identify typical SHS concentrations in broad categories of location and/or activity.

### Salivary cotinine

All prison staff were informed of our intention to gather daytime saliva samples to measure cotinine as a marker of SHS and invited to take part in providing a pre- and post-shift sample of saliva; researchers also directly recruited staff arriving for early, day, or late (but not night) shifts on the saliva sampling days. Participation was restricted to non-smokers, not using any type of nicotine product (gums, patches, e-cigarettes), and who neither lived with a smoker nor travelled to work in a vehicle where smoking took place. Consent was obtained from all participating volunteers. Saliva samples were collected using a published method similar to that used for other occupational groups (Semple *et al.*, 2007a). The exact time of sample collection was noted pre- and post-shift to calculate the exposure time. Saliva samples were stored at room temperature before shipping to ABS Laboratories, UK, for analysis. Samples were analysed for cotinine using a method employing liquid chromatography-tandem mass spectrometry (LC-MS/MS); this method had a LOD of 0.1 ng ml^−1^ and was cross-validated to the previous GC-NPD method (Feyerabend & Russell, 1990) in an inter-laboratory study (Bernert *et al.*, 2009). Where the laboratory analysis indicated a value of <LOD a value was imputed based on one-half the LOD (0.05 ng ml^−1^). This imputation method was employed to allow direct comparison with data from the Scottish Health Survey (Scottish Health Survey, 2015). Participants were excluded as likely to be a smoker or to have been heavily exposed to SHS elsewhere if they had either a pre- or post-shift saliva sample >5 ng ml^−1^. Jarvis *et al.* (2008) reported that a threshold of 5 ng ml^−1^ was optimum to discriminate between smokers and non-smokers who had no exposure to SHS at home.

Post-shift salivary cotinine data were analysed to provide an overall indication of nicotine intake among this group of workers. In addition, pre- and post-shift salivary cotinine data were used to calculate SHS exposure, expressed as a PM_2.5_ shift equivalent. Repace *et al.* (2006) developed a series of equations to link salivary cotinine changes with predicted SHS-PM concentrations. This analysis was carried out on a sub-sample of those participants who provided valid saliva samples and had both pre-shift and predicted post-shift values that were >LOD. This latter requirement is due to the need to capture information on change in salivary cotinine between the pre- and post-shift samples. Values for the toxicokinetics of cotinine elimination in the general population (Jarvis *et al.*, 1988) were applied to the pre-shift salivary cotinine value to then calculate a hypothetical post-shift value that would have occurred if the subject was not exposed to SHS while at work. The difference between this hypothetical value and their actual measured post-shift value was then calculated. A positive value is indicative of SHS exposure during the time period between samples. A worked example is provided in the online supplementary material (available at *Annals of Work Exposures and Health*).

### Statistical analysis

Data were analysed in Microsoft Excel and IBM SPSS version 24 with measures of central tendency including arithmetic means, geometric means (GMs), medians, ranges, and percentiles presented where appropriate. For the Dylos PM_2.5_ data, the percentage of time when measurements were above specific thresholds was calculated using an Excel function. As PM_2.5_ is not specific to SHS and can also arise from traffic and industrial air pollution, outdoor PM_2.5_ data were gathered from the nearest available environmental monitoring station via the website www.scottishairquality.co.uk. In the case of HMP Dumfries, the nearest environmental PM_2.5_ monitoring station was in Carlisle with data for this site taken from www.airqualityengland.co.uk. Data were extracted to match the times the in-prison measurements were made to facilitate comparison with the in-prison area PM_2.5_ measurements.

## Results

### Area monitoring

All 15 prisons in Scotland carried out the area monitoring between 30 September and 7 November 2016. Two prisons experienced problems with their Dylos device: in one the device was repeatedly switched off and in the other the power adapter failed. After data downloading revealed these problems, both establishments repeated the sampling to ensure complete data coverage. All 15 prisons gathered airborne nicotine concentrations, although data from one monitor was excluded (prison #3) because the staff member noted that it had been tampered with, resulting in a hole in the outer membrane.

Table 1 provides details of prisoner capacity and approximate number of staff in each prison. The 15 prisons in this study include a range of prison types from buildings that were first established in the 16^th^ century and subsequently modified through to several new-build establishments opened within the past decade. Most prisons have undergone recent refurbishment and some are mixed in terms of modern residential accommodation that is combined with older sections. Ventilation and heating systems vary considerably both between and within individual prisons.

**Table 1.** Prison details and results of area PM_2.5_ and nicotine measurements.

Prison details			PM_2.5_						Ambient^b^ PM_2.5_	Nicotine
Prison	Prisoner capacity	Staff numbers	Duration, minutes	% >10^a^, µg m^−3^	% >25^a^, µg m^−3^	% >246^a^, µg m^−3^	Maximum, µg m^−3^	Mean (SD), µg m^−3^	Mean (SD), µg m^−3^	µg m^−3^
P1	700	347	8635	40	16	0	64	11.2 (9.4)	6.6 (6.9)	0.349c
P2	1000	630	8644	99	93	0	222	54.6 (37.5)	11.4 (4.9)	0.592d
P3	285	112	8610	86	50	0	62	28.8 (16.7)	10.5 (3.7)	-e
P4	230	195	8638	98	78	20	1009	135.9 (189.4)	5.2 (11.4)	1.651
P5	180	162	7301	89	70	3.1	569	48.6 (62.4)	9.4 (4.1)	0.608
P6	870	476	8641	93	63	0	132	28.5 (15.8)	5.9 (2.6)	0.436
P7	670	401	8640	97	71	0	171	36 (15.1)	6.4 (2.2)	0.159
P8	500	334	8700	94	66	0	198	31.7 (16.2)	22.8 (6.2)	0.323
P9	249	198	8642	87	45	0	162	23.4 (13.5)	5.3 (1.4)	0.169
P10	103	123	8642	81	55	0.1	272	49.2 (48.6)	5.7 (2.1)	0.546
P11	500	257	8701	84	57	0.1	335	32 (20.8)	11.5 (6.0)	0.101^c^
P12	784	352	8645	68	39	0	111	19.8 (12.1)	12.6 (5.0)	0.183^d^
P13	630	368	8627	94	69	0	142	35.3 (21.0)	5.3 (1.6)	<LODf
P14	712	452	8704	87	69	0.1	466	31.1 (18.8)	6.5 (4.7)	0.319
P15	553	370	8661	40	7	0	125	10.5 (8.7)	7.7 (5.5)	<LOD^f^
Overall median				87	63	0	171	31.7	6.6	0.319

^a^These columns provide information on the percentage of 1-minute measurements that exceed each of these benchmarking values. The 10 µg m^−3^ value is the WHO air quality guidance for PM_2.5_ averaged over 1 year; the 25 µg m^−3^ value refers to the WHO guidance averaged over 24 h; the 246 µg m^−3^ value is the average value measured in Scottish pubs prior to smoke-free legislation in 2006.

^b^These are the outdoor ambient PM_2.5_ concentrations from the nearest ambient monitoring station to each prison. The median distance to these stations was 16 km (range 3–133 km).

^c^Not contemporaneous data.

^d^Average of duplicates (P2 was 0.637 and 0.546 µg m^−3^; P12 was 0.137 and 0.230 µg m^−3^).

^e^Monitor membrane damaged.

^f^Where LOD ½ the LOD was applied for calculation of summary statistics. The Dylos measurements provide detailed, time resolved data on how PM_2.5_ concentrations change over the course of each day and the 6-day measurement period. All 15 time plots are provided in the online supplementary material (available at *Annals of Work Exposures and Health*) but one example, chosen for illustration because its mean PM_2.5_ is closest to the overall median, is shown in Fig. 1.

### PM_2.5_ measurements

A total of 128431 minutes of PM_2.5_ data were collected from the 15 prisons—equivalent to over 89 days of measurement with a time resolution of 1 minute. The mean sampling duration was 8562 minutes (5.95 days, range 5.1–6.04 days).

The mean concentration for each prison was calculated, with values ranging between 11 µg m^−3^ and 136 µg m^−3^. The median of these values was 31.7 µg m^−3^.

Table 1 summarizes the measurements in each prison, including the percentage of the sampling period when concentrations were above three comparative thresholds. The first is the WHO guideline for PM_2.5_ in indoor air over a year (10 µg m^−3^); the second is the WHO guideline for PM_2.5_ in indoor air over a 24-h period (25 µg m^−3^); and the third is the average level of PM_2.5_ measured in bars in Scotland prior to smoke-free legislation in 2006 (246 µg m^−3^; Semple *et al.*, 2007b). None of these are occupational exposure limits and due to high variability when measuring exposures each minute, it is important to realise that they should not be compared directly to these guidelines but they do provide a meaningful indication of the proportion of time within each prison when PM_2.5_ levels exceed these given concentrations.

Mean ambient PM_2.5_ concentrations from the nearest local authority monitoring station were generally much lower than those measured in prisons with a median value of 6.6 µg m^−3^ (range 5.2–22.8 µg m^−3^).

**Figure 1.**
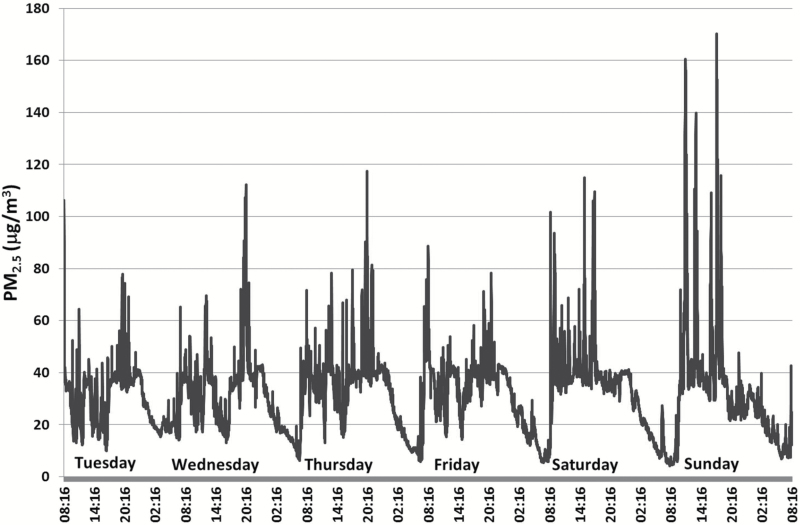
Area PM_2.5_ concentrations (1-minute measurements) measured over a 6-day period in P #8.

Fig. 1 shows a diurnal pattern, also seen in most other prisons, with higher concentrations occurring between 7 am and about 11 pm and much lower concentrations between 11 pm and 7 am. This reflects prisoner smoking activity combined with the several hours that SHS remains suspended in the air after a cigarette is smoked.

In 14 of the 15 prisons, PM_2.5_ concentrations were higher during the ‘day-time’ hours of 7 am to 11 pm. The exception was Prison #3, an ‘Open Prison’, where prisoners are allowed to leave on temporary licence to work within the community or return home for limited periods and can move more freely within the prison. Across the 15 prisons, the median value during ‘day-time’ was 36.5 µg m^−3^ compared to 20.6 µg m^−3^ for the night-time hours (11 pm–7 am), indicating that ‘day-time’ concentrations are about 80% higher than those measured during ‘night-time’ hours.

### Nicotine results

Table 1 also presents the results from the nicotine monitors. Nicotine was detected on 14 monitors (in 12 prisons) with no nicotine detectable on the filter analysed from monitors in prisons #13 and #15. In the two prisons where duplicate measurements were made, the arithmetic mean of these two samples was used, but agreement between these paired samples was variable with values differing by 8% (Prison #2) and 26% (Prison #12) from the arithmetic mean.

The median airborne concentration of nicotine measured was 0.32 µg m^−3^ (range <LOD—1.65 µg m^−3^).

### Comparison of PM_2.5_ and nicotine measurements

Data were analysed from the 12 prisons where contemporaneous measurement from both Dylos PM_2.5_ and nicotine monitors was available (i.e. excluding two prisons with non-contemporaneous nicotine measurements and one where the membrane was damaged) to determine the relationship between these measurement methods. The R-squared value of 0.91 demonstrated a very high association between the two measures. Even after excluding the datapoint from Prison #4 with the highest PM_2.5_ and nicotine concentrations, the R-squared value is 0.60.

### Mobile monitoring results

In total, 86 mobile measurements were undertaken using the Dylos devices across the 15 prisons. The median number of mobile measurements made in each prison was six (range 2–8), and total sampling duration was 2860 minutes (sample median was 30 minutes; range 5–150 minutes). Data were transformed and corrected in the same manner as the Dylos area measurements.

Each period was assigned a code based on the location where the measurement was taken or the activity taking place. Table 2 shows the median and range for each location/activity code with data pooled across all prisons. The dashed line helps demarcate locations/activities where SHS is likely to be present based on the median value >10 µg m^−3^. From the 86 mobile measurements, the lowest PM_2.5_ measurement was 0.8 µg m^−3^, the highest 753.6 µg m^−3^ and the GM (and geometric standard deviation (GSD)) was 24.1 µg m^−3^ (4.2).

**Table 2.** PM_2.5_ concentrations combined across all 15 prisons and categorised by location/activity.

Location/activity	*N*	PM_2.5_ minimum, µg m^−3^	PM_2.5_ maximum, µg m^−3^	Mean (SD) PM_2.5_, µg m^−3^	Median (IQR)^a^ PM_2.5_, µg m^−3^
Reception	5	1.0	4.1	2.6 (1.3)	2.4 (1.9–3.8)
Teaching area	6	0.8	15.5	5.4 (5.2)	4.1 (2.7–4.9)
Health care/gym	5	1.1	8.7	4.8 (2.8)	4.7 (3.4–6.0)
Outdoor	2	4.0	7.8	5.9 (2.7)	5.9 (4.0–7.8)
Staff office	5	7.8	42.9	21.9 (13.0)	18.8 (16.9–23.3)
Workshops	9	8.5	217.1	45.3 (66.6)	19.1 (11.1–46.0)
Residential corridor/landing	12	3.5	436.4	98.0 (146)	37.5 (24.7–69.6)
Cell unlocking/locking	18	4.2	89.7	40.5 (22.7)	40.4 (26.7–49.3)
Cell search/inspection	17	7.8	753.6	122 (185)	44.1 (24.1–111)
Recreation^b^	5	31.2	309.7	106 (116)	72.2 (32.6–86.7)
Cell maintenance	2	53.9	103.4	78.7 (35.0)	78.6 (53.9–103)

^a^IQR = inter-quartile range.

^b^Indoor recreation in residential areas.

All mobile measurement graphs from each prison are available in the online supplementary material (available at *Annals of Work Exposures and Health*).

### Salivary cotinine results

Saliva samples were collected from prison staff in all prisons between 7 November 2016 and 16 January 2017. In total, 422 eligible prison staff working within their prison on the day of sampling agreed to provide a sample. The median number of participants per prison was 27 (range 5–74). Three participants did not provide valid pre- or post-shift saliva samples and were excluded from further analysis. Using a threshold of >5 ng ml^−1^ at either pre- or post-shift sampling as a criterion to determine if the participant was a smoker, we excluded a further 12 participants leaving a data set of 407 subjects.

Using the post-shift value as a broad indicator of the exposure of prison staff to SHS and to provide comparison with other relevant studies, the median and GM values were calculated. The median was 0.155 (range <LOD to 1.65) ng ml^−1^ while the GM (and GSD) was 0.145 (2.48) ng ml^−1^. Table 3 provides these data by prison.

**Table 3.** Post-shift salivary cotinine concentrations from participants in all 15 prisons.

Prison	*N*	*N* (%) < LOD	25^th^ percentile (ng ml^−1^)^a^	75^th^ percentile (ng ml^−1^)^a^	Median (ng ml^−1^)^a^
P1	16	12 (75%)	0.050	0.132	0.050
P2	73	16 (22%)	0.112	0.323	0.194
P3	5	3 (60%)	0.050	0.175	0.050
P4	15	2 (13%)	0.132	0.292	0.214
P5	11	3 (27%)	0.050	0.240	0.187
P6	30	6 (20%)	0.115	0.380	0.166
P7	32	7 (22%)	0.105	0.275	0.170
P8	46	22 (48%)	0.050	0.328	0.106
P9	19	9 (47%)	0.050	0.252	0.120
P10	26	5 (19%)	0.117	0.492	0.206
P11	26	14 (54%)	0.050	0.301	0.050
P12	10	4 (40%)	0.050	0.315	0.198
P13	35	15 (43%)	0.050	0.229	0.139
P14	28	11 (39%)	0.050	0.244	0.129
P15	35	10 (29%)	0.050	0.246	0.154
All	407	139 (34%)	0.050	0.289	0.155

^a^Calculated based on *n* = 407; values <LOD were replaced by ½ LOD (0.05 ng ml^−1^).

To estimate the amount of nicotine inhaled during the work-shift, the dataset was refined to those participants where the pre-shift sample was >LOD and where the predicted post-shift cotinine value—if there was zero nicotine intake—was also >LOD. This resulted in a sub-sample of 149 subjects. A positive value indicates that SHS exposure was likely to have occurred. Overall, 138 of these 149 participants had a positive value where the post-shift salivary concentration exceeded the predicted value. At a cohort level, the median increase experienced by the 149 participants was +0.138 (range −0.875 to +1.406) ng ml^−1^.

Applying this value to a series of previously published equations (Repace *et al.*, 2006) relating salivary cotinine with PM from SHS suggests that, overall, this group of workers was exposed to an average concentration of SHS-PM of 24.8 µg m^−3^.

## Discussion

### Summary of findings and comparison with literature

To our knowledge, this is the first study to provide comprehensive evidence of prison workers’ exposure to SHS throughout a country’s entire prison system. Across a suite of measurement methods that include air sampling, biological markers of exposure, and subjective self-report, we have provided evidence of SHS exposure within cells, prison landings, halls, and other communal areas that is regular and systematic in all prisons, but varied by time of day, and between and within different prisons.

The 6-day PM_2.5_ concentrations measured in a residential hall of each prison are comparable with studies from other countries. The median value reported here was 31.7 µg m^−3^ (range 11–136 µg m^−3^) which is similar to the median value of 35.6 µg m^−3^ (range 27–70 µg m^−3^) reported from five prisons in England and Wales assessed in a near identical manner using the Dylos DC1700 device (Semple *et al.*, 2015a). Other data from four prisons in England (Jayes *et al.*, 2016) used a TSI Sidepak AM510 to measure PM_2.5_ concentrations over shorter periods (mean 6.5 hours) on residential landings and reported average concentrations of 43.9 µg m^−3^ on wings where smoking within cells was permitted. Given that Jayes *et al.*’s data were gathered during ‘daytime hours’, it is worth noting that the 6-day residential hall results from the present study were 36.5 µg m^−3^ when restricted to daytime hours.

Studies from prisons in other parts of the world provide more divergent results. A study in a single New Zealand prison (Thornley *et al.*, 2013) used the TSI Sidepak AM510 to measure PM_2.5_ concentrations before the introduction of a tobacco ban and reported a GM value of 6.6 µg m^−3^ over a 14-day period. The device was positioned in the staff base adjacent to the four prison wings. Previous work examining PM_2.5_, again with the TSI Sidepak AM510, in six prisons in the USA (Proescholdbell *et al.*, 2008) provided mean values of 93.1 µg m^−3^ from measurements in prison dormitory areas and lobbies. These 14 measurements were taken over short periods with between 43 and 91 minutes spent in each of the six prisons. A study in a Swiss prison (Ritter *et al.*, 2012) reported PM_10_ concentrations made in three prison areas with mean values of 30, 120, and 180 µg m^−3^, however, duration of measurement was not reported.

The 31.7 µg m^−3^ PM_2.5_ median in the current study can also be compared to other smoking and smoke-free environments. For context, the average values reported for smoke-free homes in Scotland is 3.1 µg m^−3^ (Semple *et al.*, 2015c). Smokers’ homes in Scotland have a median value of 31 µg m^−3^ (Semple *et al.*, 2015c)—very similar to the 6-day area value measured across the 15 Scottish prisons in this study. Data on PM_2.5_ concentrations measured in Scottish pubs and bars prior to smoke-free legislation in 2006 indicated a mean value of 246 µg m^−3^ (Semple *et al.*, 2007b), nearly eight times greater than that measured in Scottish prisons.

The GM for the 86 mobile PM_2.5_ measurements was 24.1 µg m^−3^ (GSD 4.2), very similar to that for 70 ‘spot’ measurements using a near identical protocol in six prisons in England and Wales (GM 24 µg m^−3^; GSD 3.5) in 2015 (Semple *et al.*, 2015a). Time-course graphs of both the area and mobile monitoring results show the wide range of PM_2.5_ concentrations measured, by prison, time of day and specific locations and activities. The mobile measurement results suggest that some areas of most prisons, including health care, sports/gym facilities, teaching, and reception areas, are essentially smoke-free. Many workshop area measurements also indicate little, if any, SHS exposure. However, staff exposure is considerable in many other areas, particularly those close to cells. Staff offices, corridors, and landings show evidence of SHS drifting from prisoners’ cells to these communal areas. Concentrations during recreation activities were particularly high. Activities involving cell unlocking, cell searches, cell fabric inspections, and cell maintenance generally suggest considerable exposure. These activities may result in staff being exposed to concentrations that are several times higher than the WHO guideline for PM_2.5_ with some of these activity-based measurements indicating values comparable with those measured in Scottish bars when smoking was permitted (Semple *et al.*, 2007b).

The airborne nicotine measurements reported in this study had a median of 0.32 µg m^−3^. These values are considerably lower than we would have anticipated given the PM_2.5_ results from the co-located Dylos DC1700 devices together with the data on likely nicotine concentrations from saliva samples.

We note that the ‘Rosetta stone’ equations developed by Repace and colleagues (2006) suggest that PM_2.5_ concentrations are roughly 10 times those of airborne nicotine in settings where SHS is present. Given the Dylos median of 31.7 µg m^−3^, we would anticipate an air nicotine median of about 3.2 µg m^−3^. In comparison, Hammond and Emmons (2005) measured weekly airborne nicotine concentrations in three US prisons before smoke-free rules were put in place. Their analysis of 84 locations indicated average values ranging between 3 and 11 µg m^−3^ in most living and sleeping areas within these prisons. Ritter *et al.* (2011) reported mean values of 7.0 µg m^−3^ in a Swiss prison, while work in smoking homes by Phillips and co-workers (1996) and by Butz *et al.* (2011) indicated airborne nicotine concentrations of 1.1 and 1.4 µg m^−3^, respectively. Both these studies (Butz *et al.*, 2011; Ritter *et al.*, 2011) also reported PM concentrations very similar to those measured by our Dylos DC1700 devices in prisons (39 and 35 versus 32 µg m^−3^). Our results using pre- and post-shift cotinine also suggest that prison workers’ nicotine intake matches with the 20–30 µg m^−3^ estimate of PM_2.5_ when using the Repace (2006) Rosetta Stone equations.

There are two possible explanations for the low concentrations of airborne nicotine we measured: firstly it is possible that the nicotine results we report are correct given that they were collected using a validated method; alternatively, it is possible that some systematic loss of nicotine occurred during the storage, transportation, or analysis of the filters. While we acknowledge the possibility of the former, we consider that the latter is more plausible given the evidence of SHS exposure that we report here and the lack of alternative sources of the PM_2.5_ measured. We also note a strong and consistent relationship (R-squared = 0.91) between the airborne nicotine values and the PM_2.5_ concentrations suggesting that the measured PM_2.5_ was reflecting particle emissions that were linked to SHS. After extensive discussions with the laboratory to explore potential reasons for the low nicotine results, we identified that, for a week prior to shipping to the USA, the nicotine monitors were stored in a laboratory where temperatures regularly exceeded 27°C. We are also unaware of the environmental conditions in terms of temperature and pressure that the filters may have experienced during airfreight transport. We postulate that there may have been some systematic nicotine loss from the filters during either storage and/or air transportation to the USA after collection that resulted in a systematic error. Future work should aim to collect and analyse spiked samples to examine if such losses occur and calculate recovery efficiencies for this methodology.

The high level of agreement between the Dylos measured PM_2.5_ results and the nicotine concentrations suggest that real-time measurement of PM_2.5_ with these low-cost devices presents considerable advantages over nicotine monitoring. The information on temporal changes of SHS concentrations, coupled with the simplicity of data collection with no laboratory analysis costs, provide significant practical benefits for future work in this area.

The salivary cotinine data taken at the end of the work-shift indicates a GM (GSD) value of 0.15 (2.48) ng ml^−1^. This compares to a GM (GSD) of 0.12 (3.39) ng ml^−1^ in 54 prison workers in England and Wales in 2015 (Semple *et al.*, 2015a). A salivary cotinine GM of 0.09 ng ml^−1^ was reported in the most recent (2014/5) population-level survey of non-smoking adults in Scotland (Scottish Health Survey, 2015), while historically, the GM (GSD) value measured in bar workers in Scotland prior to smoke-free legislation in 2006 was 2.94 (2.28) ng ml^−1^ (Semple *et al.*, 2007a). These data indicate that prison staff have exposure that is markedly higher than the general adult non-smoking population in Scotland, but also suggest that prison workers experience much lower exposures than those of bar workers prior to smoke-free legislation in 2006.

Using the difference between the pre- and post-shift saliva samples, we utilized Repace and colleagues’ (2006) ‘Rosetta Stone’ equations to estimate a PM equivalent exposure during the work-shift. The median increase in salivary cotinine for the 149 non-smoking workers to whom we could apply this method was 0.138 ng ml^−1^; this equates to a work-shift average of SHS-PM of 24.8 µg m^−3^. We acknowledge that this method excludes over 60% of those non-smoking prison staff who arrived at work with salivary cotinine levels <LOD and so may not be representative of the exposure of all prison workers. However, we note that the results generated by this approach are broadly in agreement with the personal PM_2.5_ measurements made on 22 prison staff in England monitored for an average of 4.2 hours (Jayes *et al.*, 2016) where a mean value of 23.5 µg m^−3^ was reported, and a study of six English prisons (Semple *et al.*, 2015a) where the GM (GSD) personal exposure of 30 prison staff to PM_2.5_ was 19 (2.2) µg m^−3^.

### Strengths and weaknesses

The number of methods used to measure prison workers’ exposure to SHS provides an opportunity to examine agreement and consider advantages and disadvantages of each approach. Prisons are a unique occupational environment that pose many challenges when measuring workers’ exposure to SHS. There is a need to consider the safety of personnel. For example, carrying out personal exposure monitoring where workers wear pumps with flexible tubing attached to their breathing zone while carrying out their operational duties, would raise important safety issues.

Prison staff assisted with the air quality data collection, after attending a half-day training session, and installed the Dylos DC1700 devices and nicotine monitors according to a standard protocol. With support by telephone and email in near real-time, the data collection was completed with minimal technical or logistic problems. This was an extremely effective use of study resources and enabled all area air quality data to be gathered in a period of just 5 weeks. We acknowledge though that prison staff had to make pragmatic decisions about which residential hall to measure in each prison, and therefore prison values may not reflect exposures in all residential halls in any prison. The collection of saliva samples was more problematic and much more resource-intensive with a team of researchers required on site for the arrival (commencing often prior to c5.45 am) and departure (up to c9.30 pm) of shifts during the day; remote prison sites required overnight travel and accommodation.

Presenting data on SHS exposure in terms of PM_2.5_ is a well-recognized method that has been employed in many studies including bars (Semple *et al.*, 2007a), homes (Semple *et al.*, 2015c), workplaces, and prisons (Jayes *et al.*, 2016). The method, however, lacks specificity to SHS and results can be elevated from non-SHS sources, such as ambient air pollution and processes such as wood/metal working. The study also examined the ambient PM_2.5_ concentrations from the nearest local government environmental monitoring station (median outdoor concentration 6.6 µg m^−3^) which provided further evidence that the values measured within prisons are from SHS sources. While we acknowledge that some prisons were located a considerable distance from the nearest ambient monitoring station the data do indicate that PM_2.5_ concentrations inside prisons were considerably higher than the levels measured over the same time period at outdoor monitoring sites. This was the case for all 15 prisons.

Presenting the results in a meaningful way to stakeholders including staff, employer representatives, and policy makers, also provides challenges given the lack of a Workplace Exposure Limit. In line with other research on SHS, we employed the WHO Air Quality Guidance Values for PM_2.5_ in indoor air though we note that the same guidance also cautions that ‘the WHO guidelines for environmental tobacco smoke (ETS) [SHS] published in the second edition of Air quality guidelines for Europe, stating that there is no evidence for a safe exposure level, are clear and still valid. Therefore, ETS [SHS] is not included in the current [guidelines]. Furthermore, the guidelines for other pollutants [i.e. PM_2.5_] should be developed based on the assumption that ETS [SHS] is eliminated from indoor spaces’ (WHO, 2010). In other words, the WHO guidance does not specifically apply to SHS as the WHO indicate that there is no safe level of exposure. Given this lack of suitable guidance it is also useful to present comparative measurement data and in this study we opted for measurements of PM_2.5_ from other prisons, smokers’ homes and from Scottish bars prior to smoke-free legislation.

It is difficult to quantify the risks to health that the SHS exposures measured in this study constitute. The risk to health for any given worker will be a function of current and past exposure to SHS, in addition to their own smoking history. Societal changes and the anecdotal reports from prison staff would suggest that exposures in the past were much higher than those measured today. Simply taking the results from the measurements made in 2016/7 and reported here, the risks from SHS are clearly much lower than those experienced by bar workers prior to smoke-free legislation in 2006. Our data suggest the exposure of a typical prison worker to SHS is broadly similar to that of a non-smoking adult living with a smoker who smokes at home. Epidemiological data on non-smokers who live with smokers (Hole, 2004) would suggest relative risk ratios of between 1.1 and 1.3 for mortality from four common diseases: lung cancer, stroke, ischaemic heart disease, and chronic obstructive pulmonary disease.

This study constitutes the most comprehensive evaluation of prison staff exposure to SHS in any country to date. There is evidence that the different methods applied are broadly in agreement with each other, with the clear exception of the data on air nicotine concentrations. The 6-day Dylos DC1700 PM_2.5_ measurements within the main hall provide a useful overview of SHS concentrations within each prison and, when married to short-duration mobile sampling from locations and activities, help give a clear picture of prison workers’ exposure to SHS. The low-cost and practical advantages of such an approach should be considered when designing other studies to evaluate the introduction of smoke-free prisons globally.

## Conclusions and Policy Implications

Staff in Scotland’s prisons are exposed to SHS. Exposures vary by prison, time of day, and the activities each staff member undertakes. Considering all the evidence available from this study, it is likely that a figure for median exposure lies somewhere between 20 µg m^−3^ and 30 µg m^−3^ of SHS-PM_2.5_; though some staff in particular prisons and during particular activities may experience exposures considerably greater than this. While there is no UK Workplace Exposure Limit or international equivalent for SHS-PM_2.5_, it is noteworthy that the WHO state that ‘there is no safe level of exposure to SHS’. For comparison purposes, this level of 20–30 µg m^−3^ SHS exposure is similar to that measured in smokers’ homes in Scotland (Semple *et al.*, 2015c).

This study provides a comprehensive evaluation of prison staff exposure across all the prisons in one country. It demonstrates that differing approaches to the assessment of exposure are required to fully understand SHS concentrations within these complex settings. The paper further illustrates the potential to use low-cost air quality monitors, installed by local prison staff trained to standard protocols, to gather high-quality exposure data in a resource-effective manner. Future work should consider this method for studies in prisons and other settings.

SHS is harmful and the vast majority of people in the UK, and many other countries, are now protected from exposure to this hazard while at work. Prison staff are aware of the protection afforded to other worker groups and see governmental campaigns and health advice on the importance of living in a smoke-free environment (Scottish Government, 2015). There is an urgent need to find suitable and effective policies that can help protect prison workers from exposure to SHS given the high proportion of prisoners who smoke.

## Supplementary Data

Supplementary data are available at *Annals of Work Exposures and Health* online.

## Conflict of Interest Declaration

The authors declare no conflict of interest relating to the material presented in this Article.

## Department of Health Disclaimer

The views and opinions expressed are those of the authors and do not necessarily reflect those of the Public Health Research Programme, NIHR, NHS, or the Department of Health.

## Supplementary Material

Suppl Material-1

Suppl Material-2_salivary_cotinine_calculation_final
