# Supplementary material for: Characterising the Exposure of Prison Staff to Second-Hand Tobacco Smoke
Source: Ann Work Expo Health. 2017 Jul 16;61(7):809–21. doi: 10.1093/annweh/wxx058 (PMC6684365; doi:10.1093/annweh/wxx058)
Supplement: Suppl Material-1 [file wxx058_suppl_suppl_material_1.pdf]

Characterising the exposure of prison staff to second-hand tobacco smoke

Semple S[1], Sweeting H[2], Demou E[2], Logan G[2], O'Donnell R[1], Hunt K[2] on behalf of the Tobacco in Prisons (TIPs) research team

[1]Respiratory Group, Division of Applied Health Sciences, University of Aberdeen, Aberdeen, AB25 2ZG, Scotland.

[2]MRC/CSO Social and Public Health Sciences Unit, Institute of Health and Wellbeing, University of Glasgow, 200 Renfield St, G2 3QB.

Supplementary material

Static monitoring data graphical outputs

Prison 1

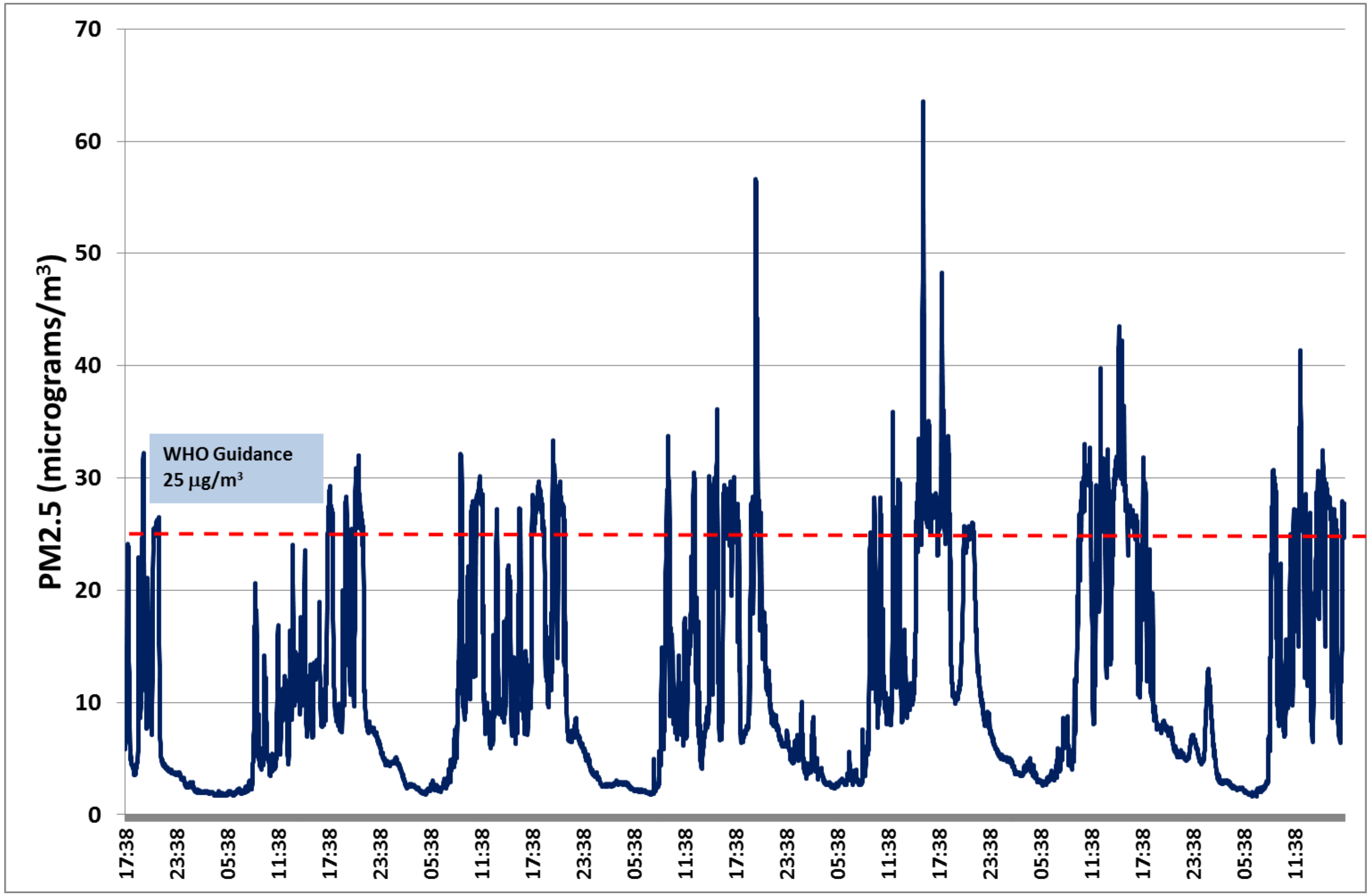

Prison 2

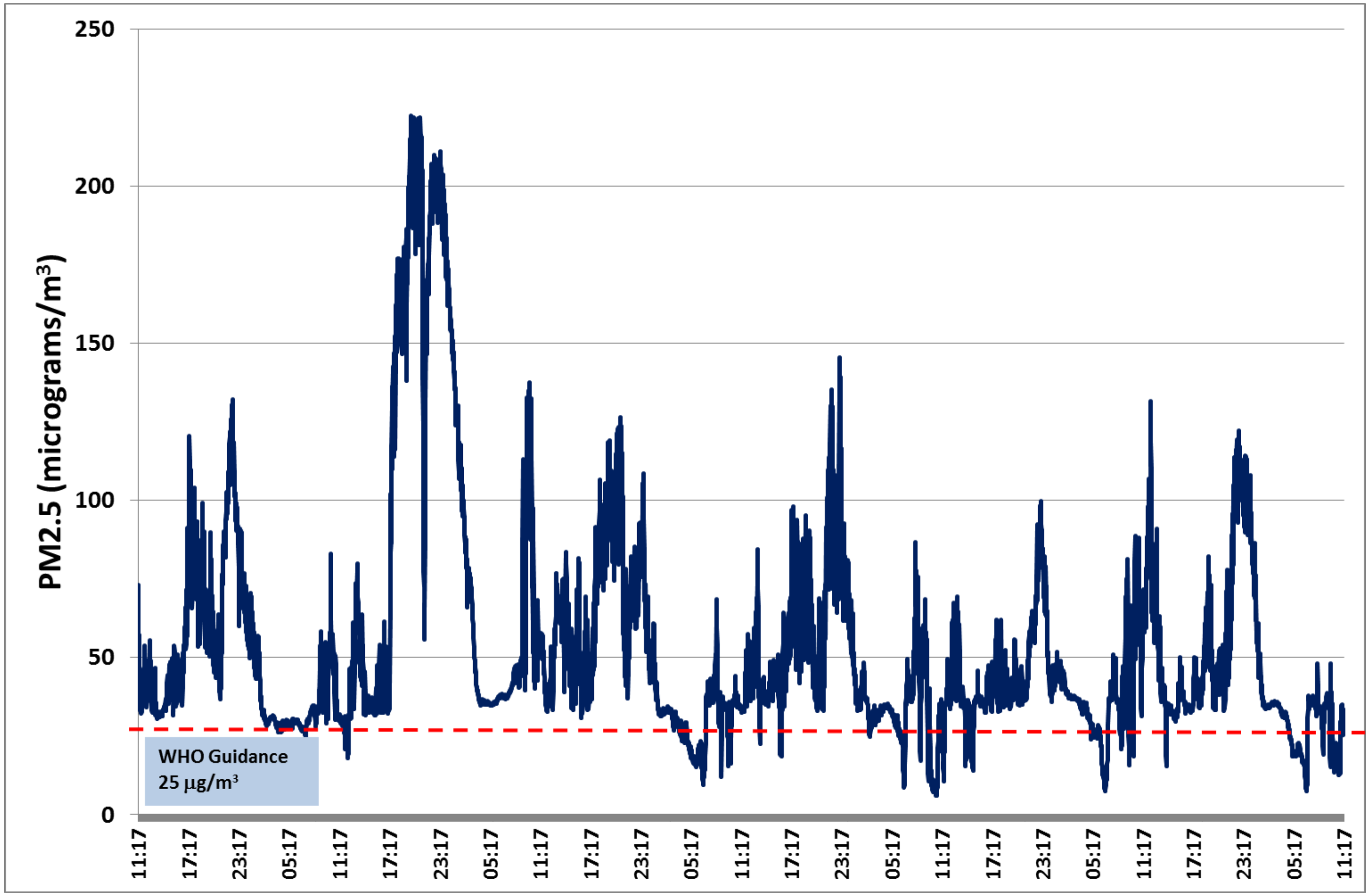

PM2.5 (micrograms/m3)
250
200
150
100
50
0
WHO Guidance
25 µg/m³
11:17
17:17
23:17
05:17
11:17
17:17
23:17
05:17
11:17
17:17
23:17
05:17
11:17
17:17
23:17
05:17
11:17
17:17
23:17
05:17
11:17
17:17
23:17
05:17
11:17

Prison 3

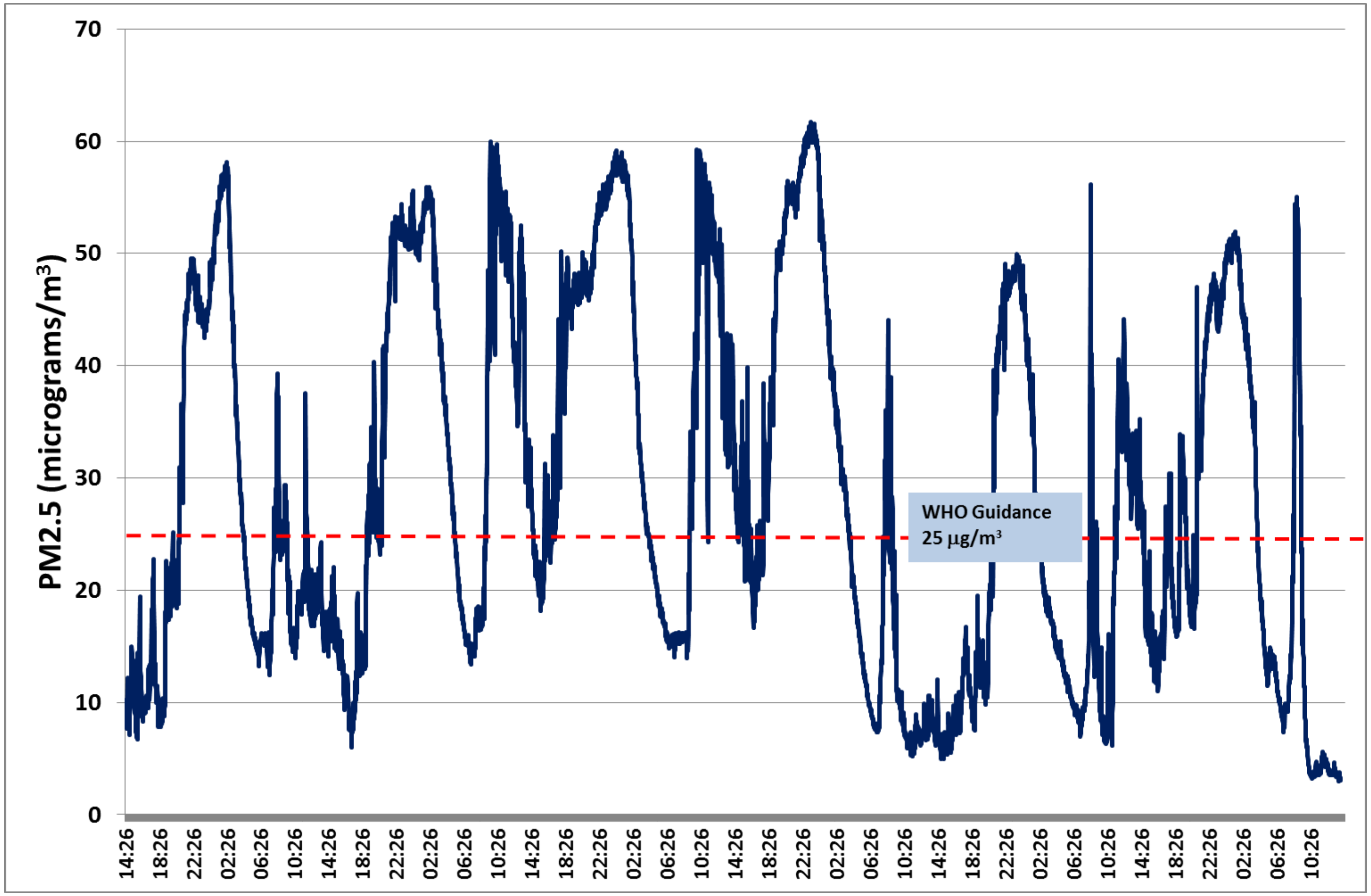

PM2.5 (micrograms/m3)
70
60
50
40
30
20
10
0
WHO Guidance
25 μg/m3
14:26
18:26
22:26
02:26
06:26
10:26
14:26
18:26
22:26
02:26
06:26
10:26
14:26
18:26
22:26
02:26
06:26
10:26
14:26
18:26
22:26
02:26
06:26
10:26
14:26
18:26
22:26
02:26
06:26
10:26
14:26
18:26
22:26
02:26
06:26
10:26

Prison 4

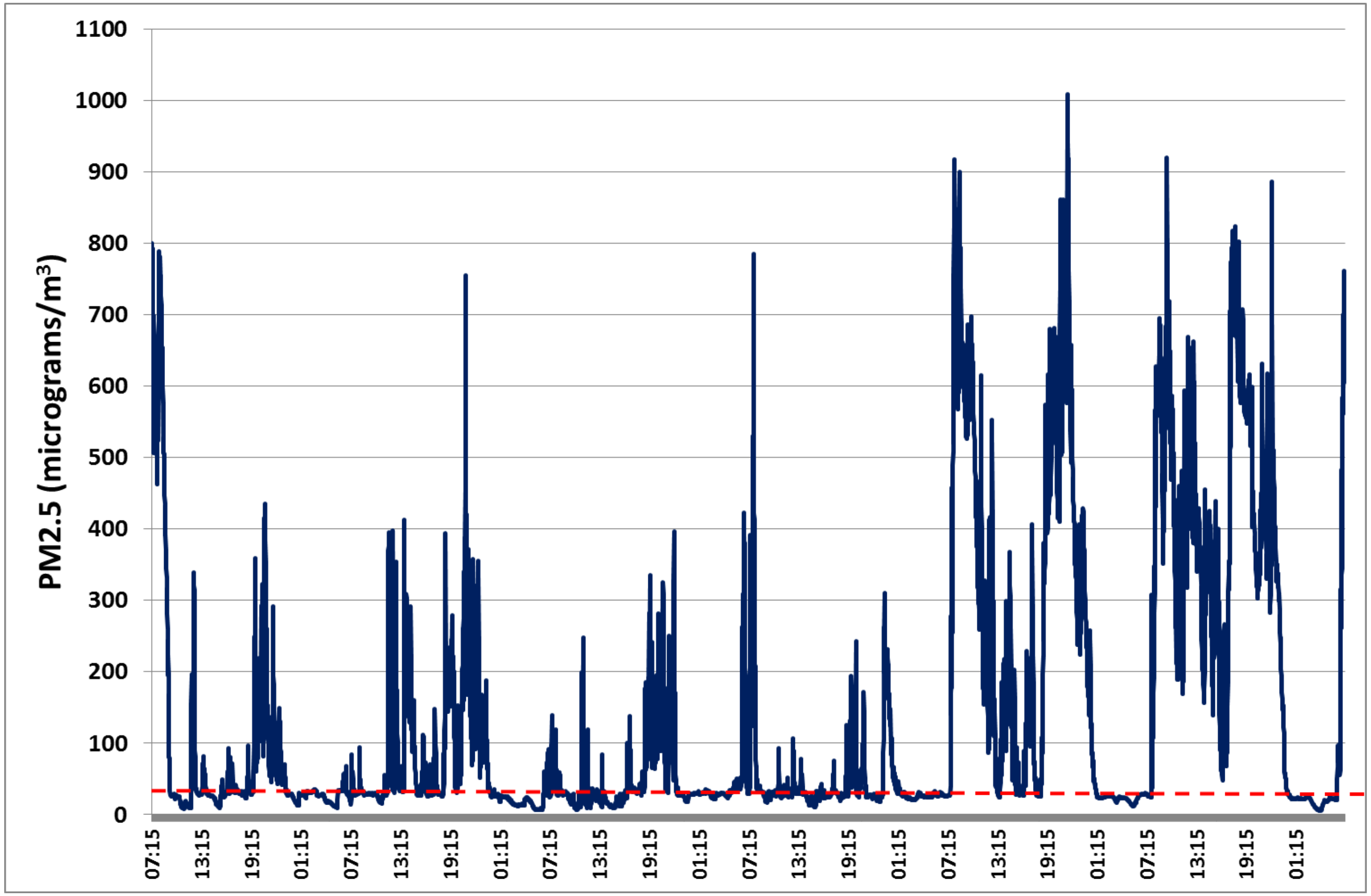

1100
1000
900
800
700
600
500
400
300
200
100
0
PM2.5 (micrograms/m3)
07:15
13:15
19:15
01:15
07:15
13:15
19:15
01:15
07:15
13:15
19:15
01:15
07:15
13:15
19:15
01:15
07:15
13:15
19:15
01:15

Prison 5

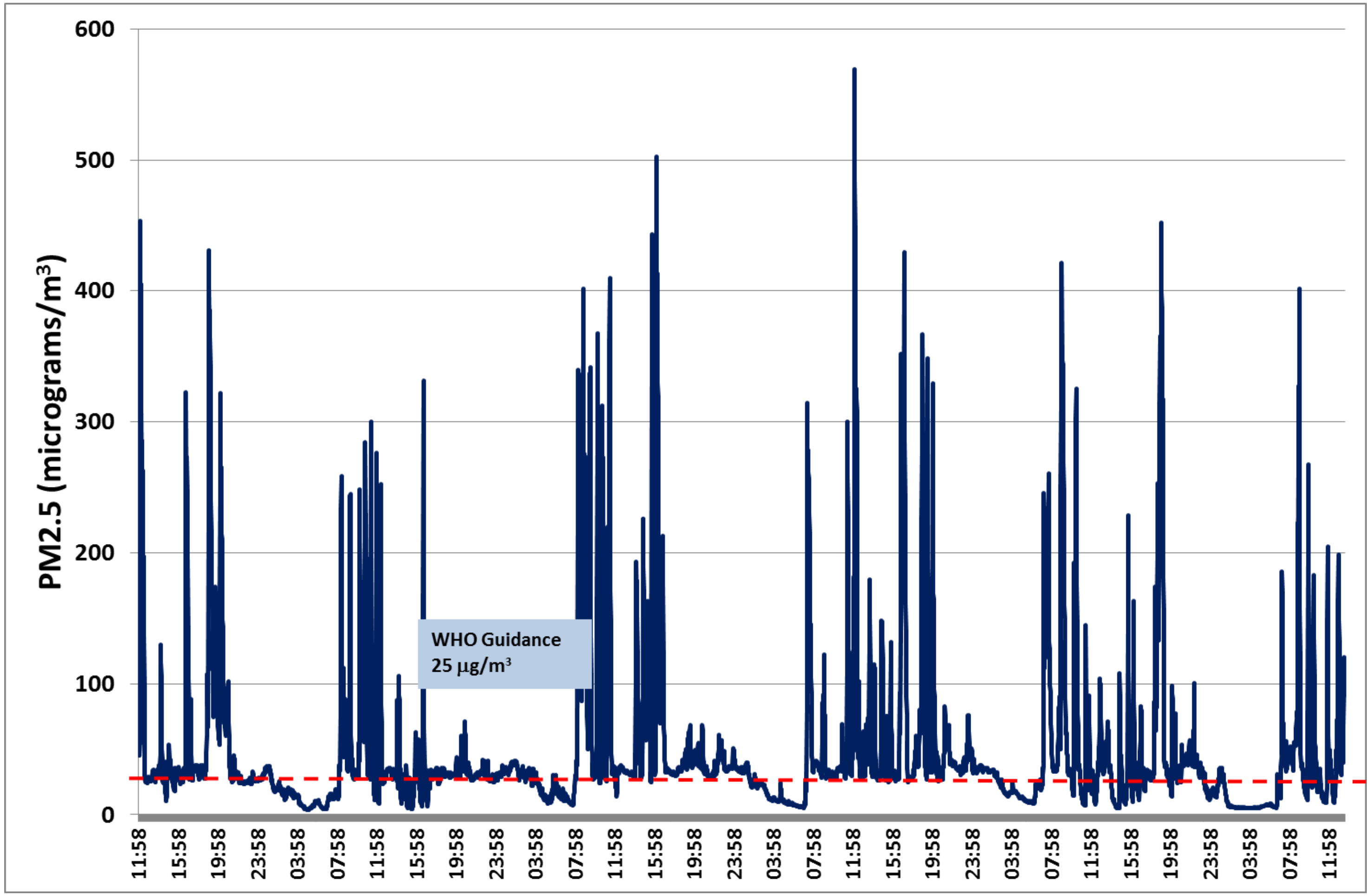

PM2.5 (micrograms/m3)
600
500
400
300
200
100
0
WHO Guidance
25 μg/m3
11:58
15:58
19:58
23:58
03:58
07:58
11:58
15:58
19:58
23:58
03:58
07:58
11:58
15:58
19:58
23:58
03:58
07:58
11:58
15:58
19:58
23:58
03:58
07:58
11:58
15:58
19:58
23:58
03:58
07:58
11:58

Prison 6

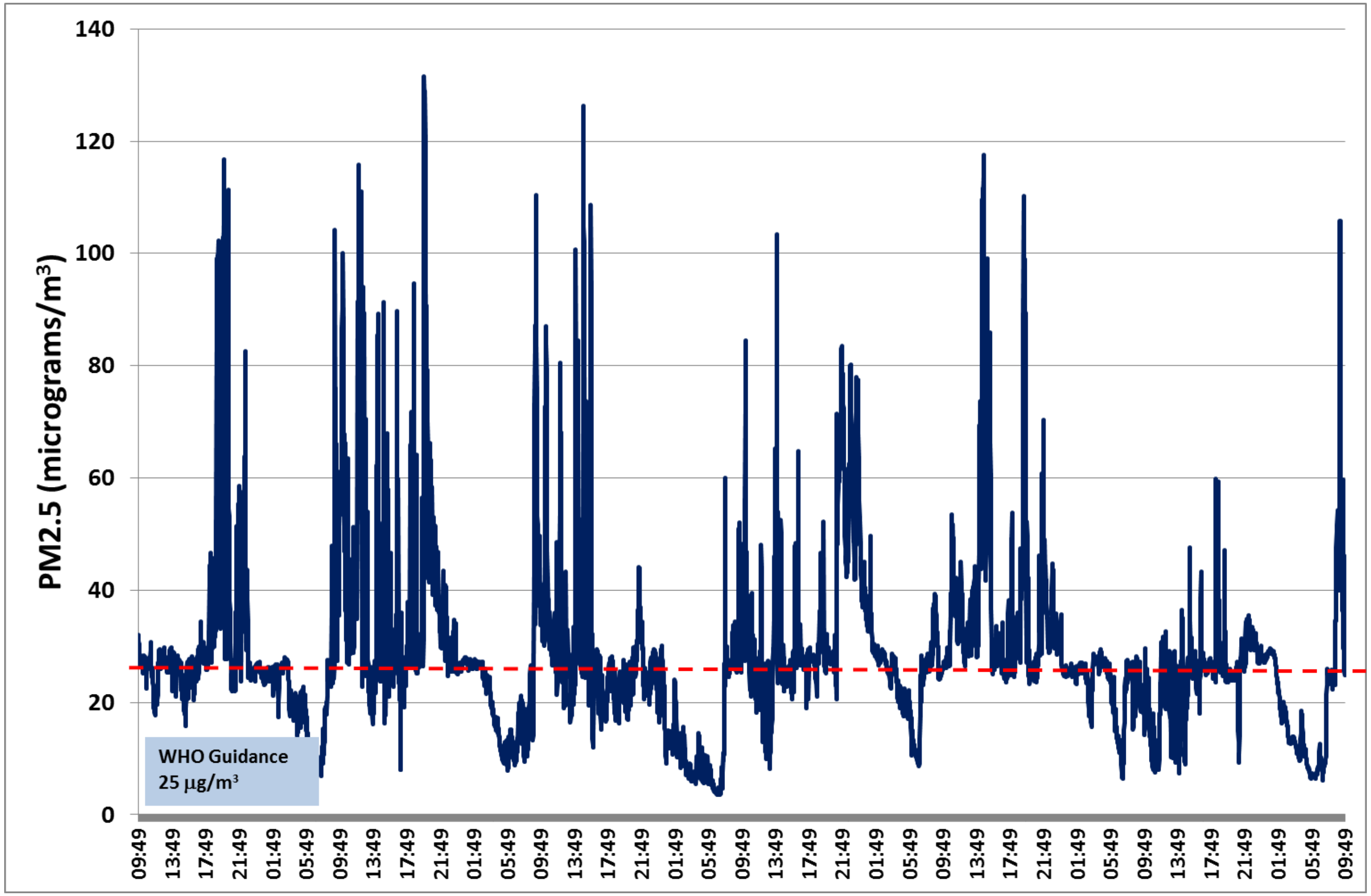

PM2.5 (micrograms/m3)
140
120
100
80
60
40
20
0
WHO Guidance
25 µg/m3
09:49
13:49
17:49
21:49
01:49
05:49
09:49
13:49
17:49
21:49
01:49
05:49
09:49
13:49
17:49
21:49
01:49
05:49
09:49
13:49
17:49
21:49
01:49
05:49
09:49
13:49
17:49
21:49
01:49
05:49
09:49
13:49
17:49
21:49
01:49
05:49
09:49

Prison 7

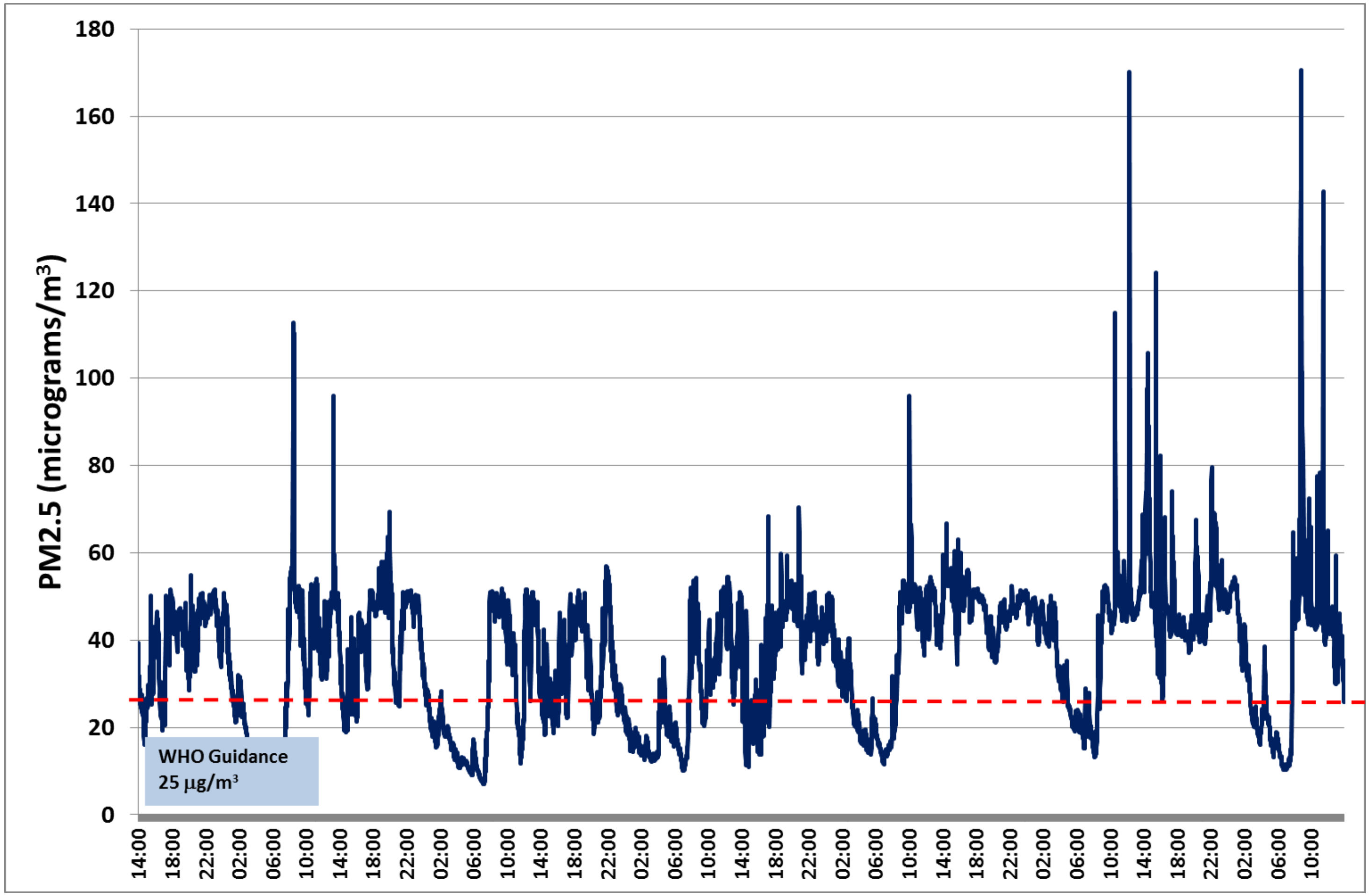
PM2.5 (micrograms/m3)
180
160
140
120
100
80
60
40
20
0
WHO Guidance
25 µg/m3
14:00
18:00
22:00
02:00
06:00
10:00
14:00
18:00
22:00
02:00
06:00
10:00
14:00
18:00
22:00
02:00
06:00
10:00
14:00
18:00
22:00
02:00
06:00
10:00
14:00
18:00
22:00
02:00
06:00
10:00
14:00
18:00
22:00
02:00
06:00
10:00

Prison 8

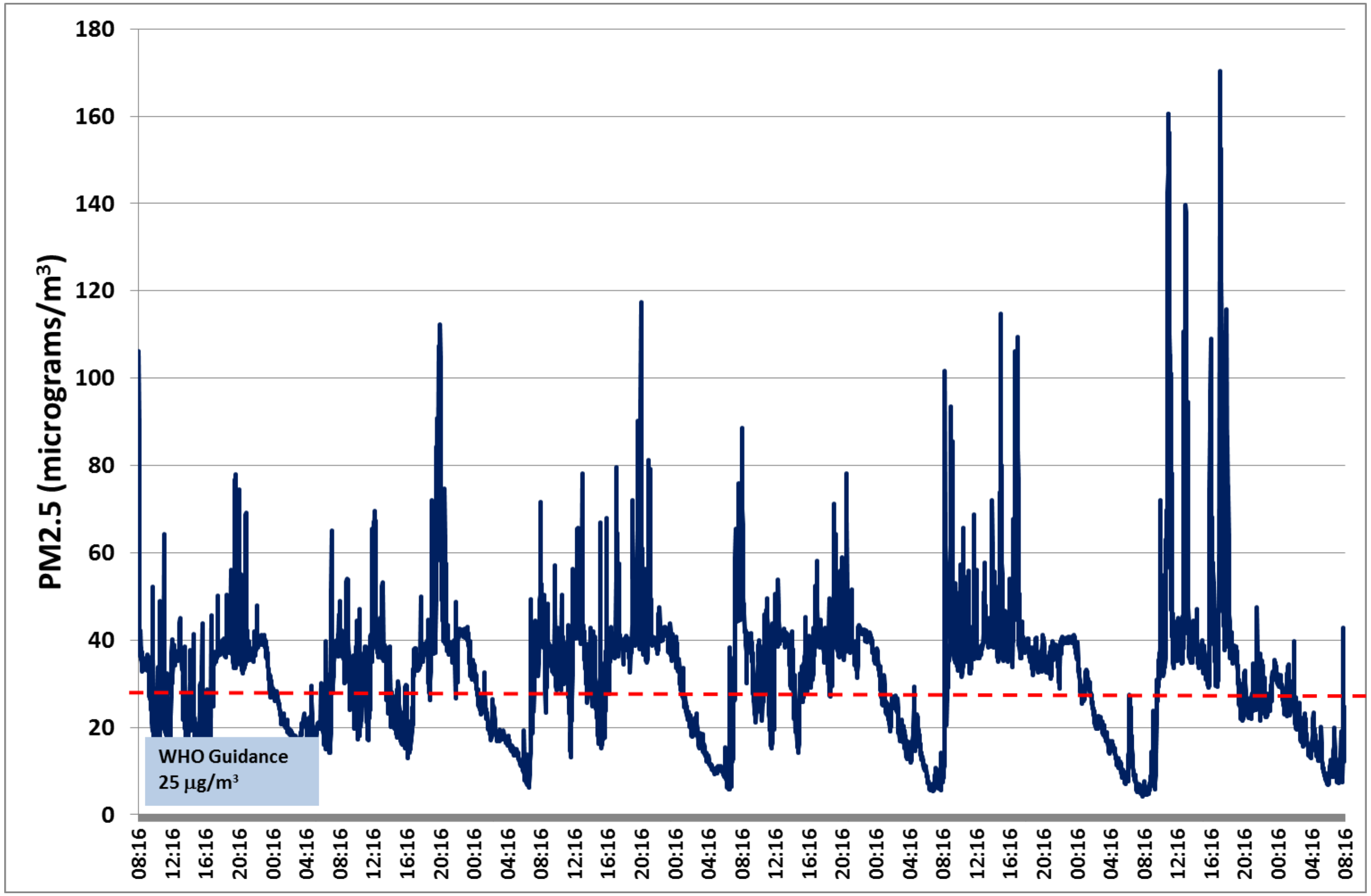
PM2.5 (micrograms/m³)
180
160
140
120
100
80
60
40
20
0
WHO Guidance
25 µg/m³
08:16
12:16
16:16
20:16
00:16
04:16
08:16
12:16
16:16
20:16
00:16
04:16
08:16
12:16
16:16
20:16
00:16
04:16
08:16
12:16
16:16
20:16
00:16
04:16
08:16
12:16
16:16
20:16
00:16
04:16
08:16
12:16
16:16
20:16
00:16
04:16
08:16

Prison 9

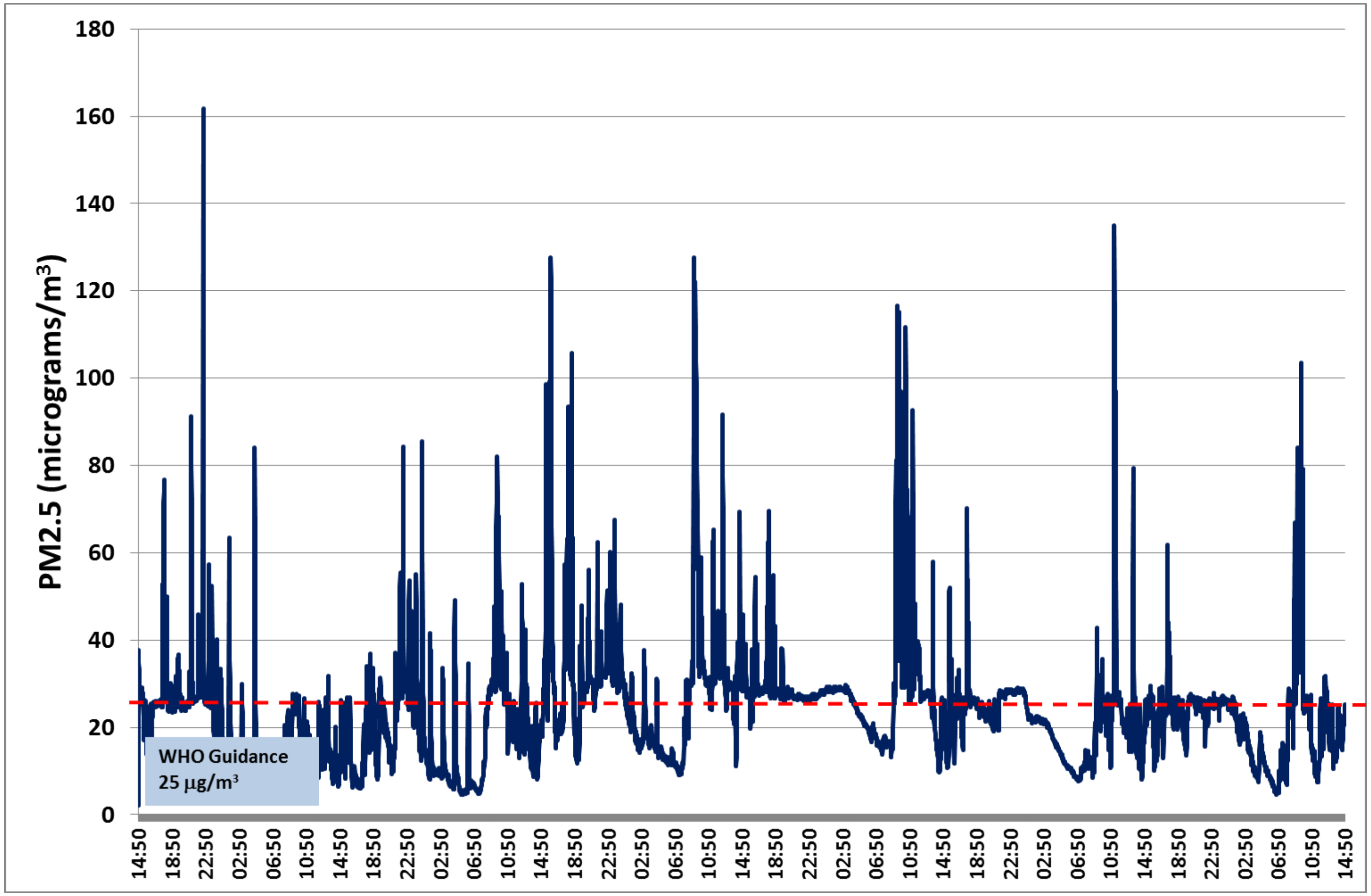
PM2.5 (micrograms/m3)
180
160
140
120
100
80
60
40
20
0
WHO Guidance
25 µg/m3
14:50
18:50
22:50
02:50
06:50
10:50
14:50
18:50
22:50
02:50
06:50
10:50
14:50
18:50
22:50
02:50
06:50
10:50
14:50
18:50
22:50
02:50
06:50
10:50
14:50
18:50
22:50
02:50
06:50
10:50
14:50
18:50
22:50
02:50
06:50
10:50
14:50

Prison 10

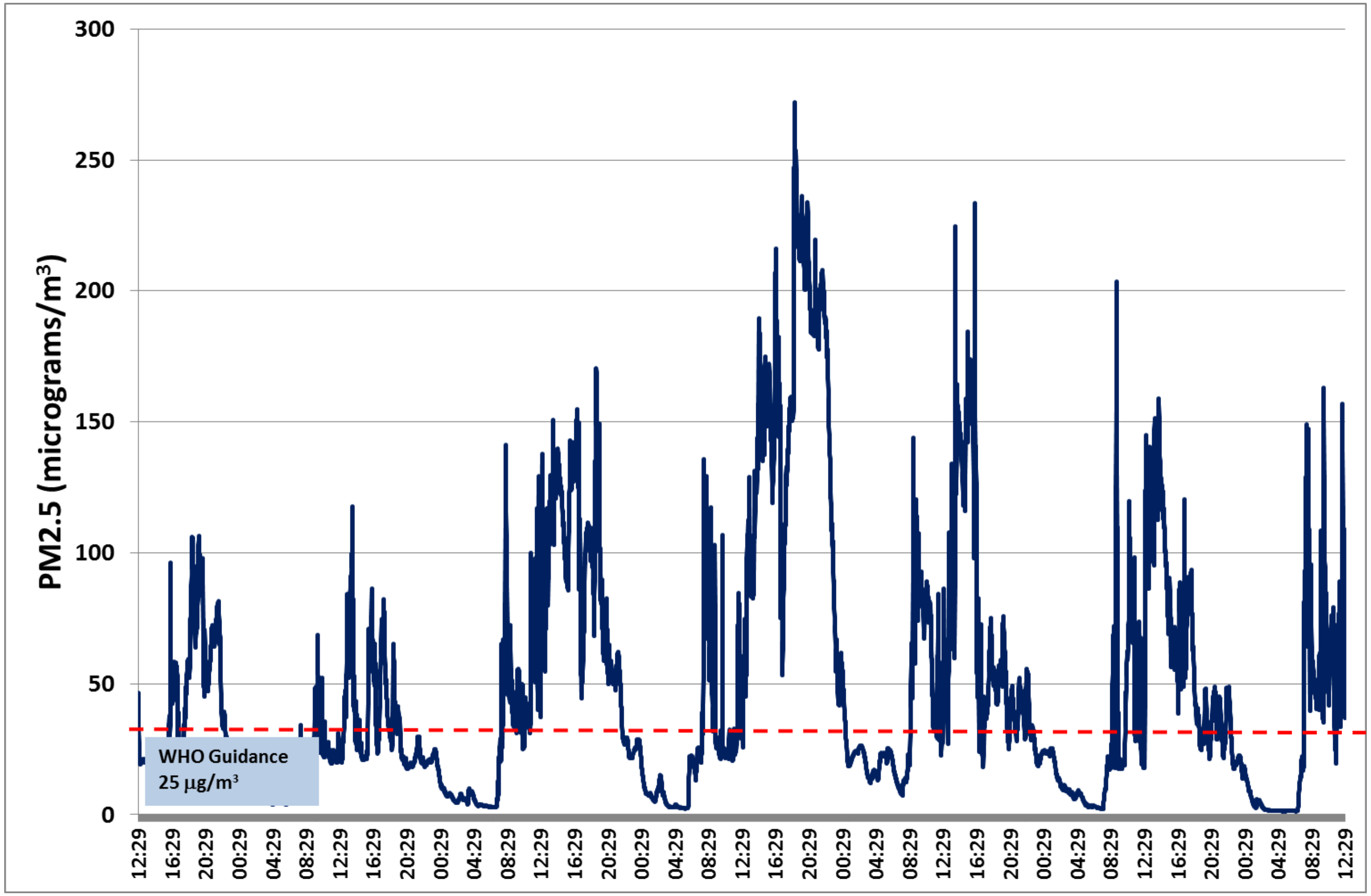

PM2.5 (micrograms/m³)
300
250
200
150
100
50
0
WHO Guidance
25 µg/m³
12:29
16:29
20:29
00:29
04:29
08:29
12:29
16:29
20:29
00:29
04:29
08:29
12:29
16:29
20:29
00:29
04:29
08:29
12:29
16:29
20:29
00:29
04:29
08:29
12:29
16:29
20:29
00:29
04:29
08:29
12:29
16:29
20:29
00:29
04:29
08:29
12:29

Prison 11

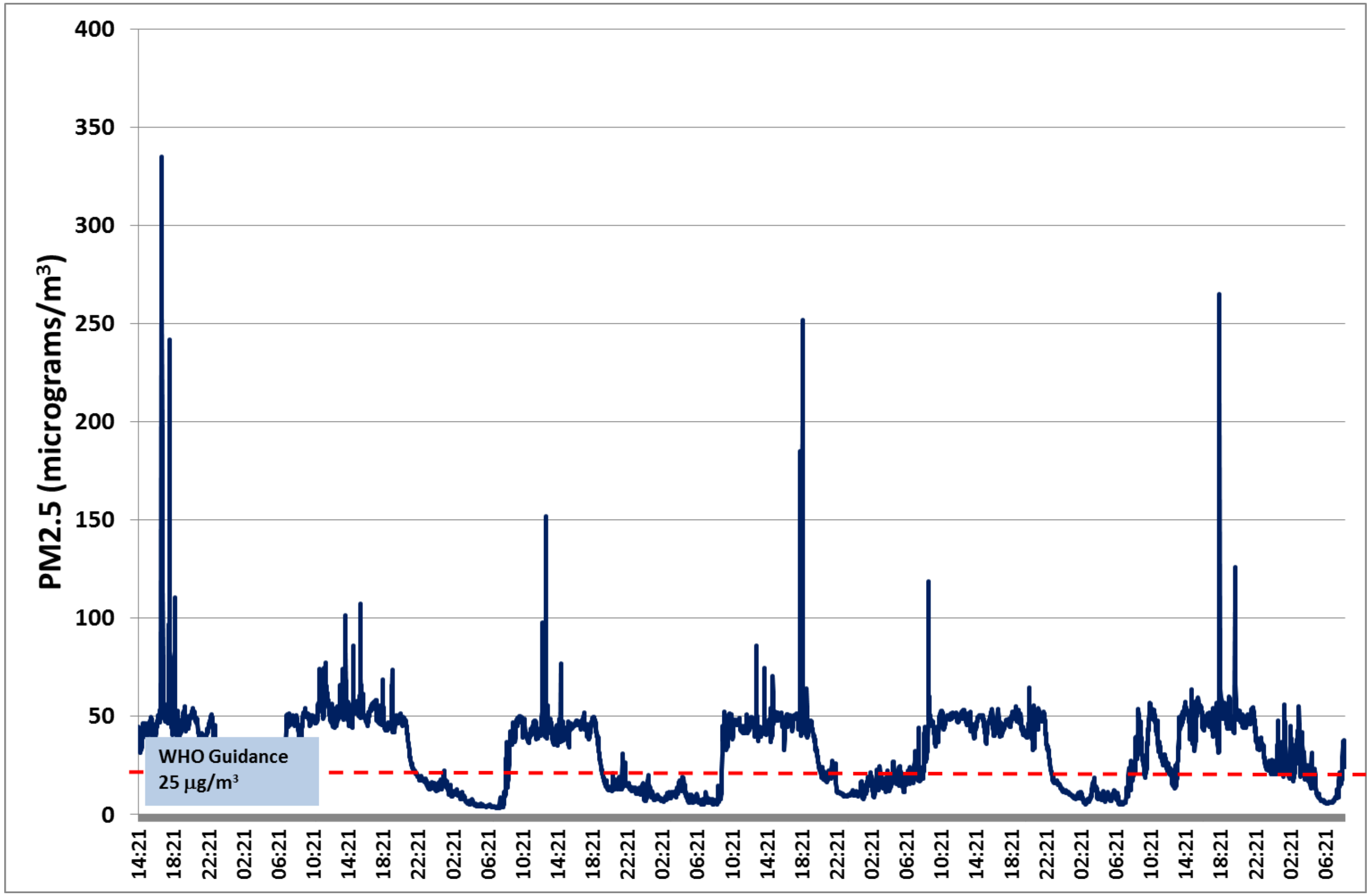

PM2.5 (micrograms/m³)
400
350
300
250
200
150
100
50
0
WHO Guidance
25 µg/m³
14:21
18:21
22:21
02:21
06:21
10:21
14:21
18:21
22:21
02:21
06:21
10:21
14:21
18:21
22:21
02:21
06:21
10:21
14:21
18:21
22:21
02:21
06:21
10:21
14:21
18:21
22:21
02:21
06:21
10:21
14:21
18:21
22:21
02:21
06:21

Prison 12

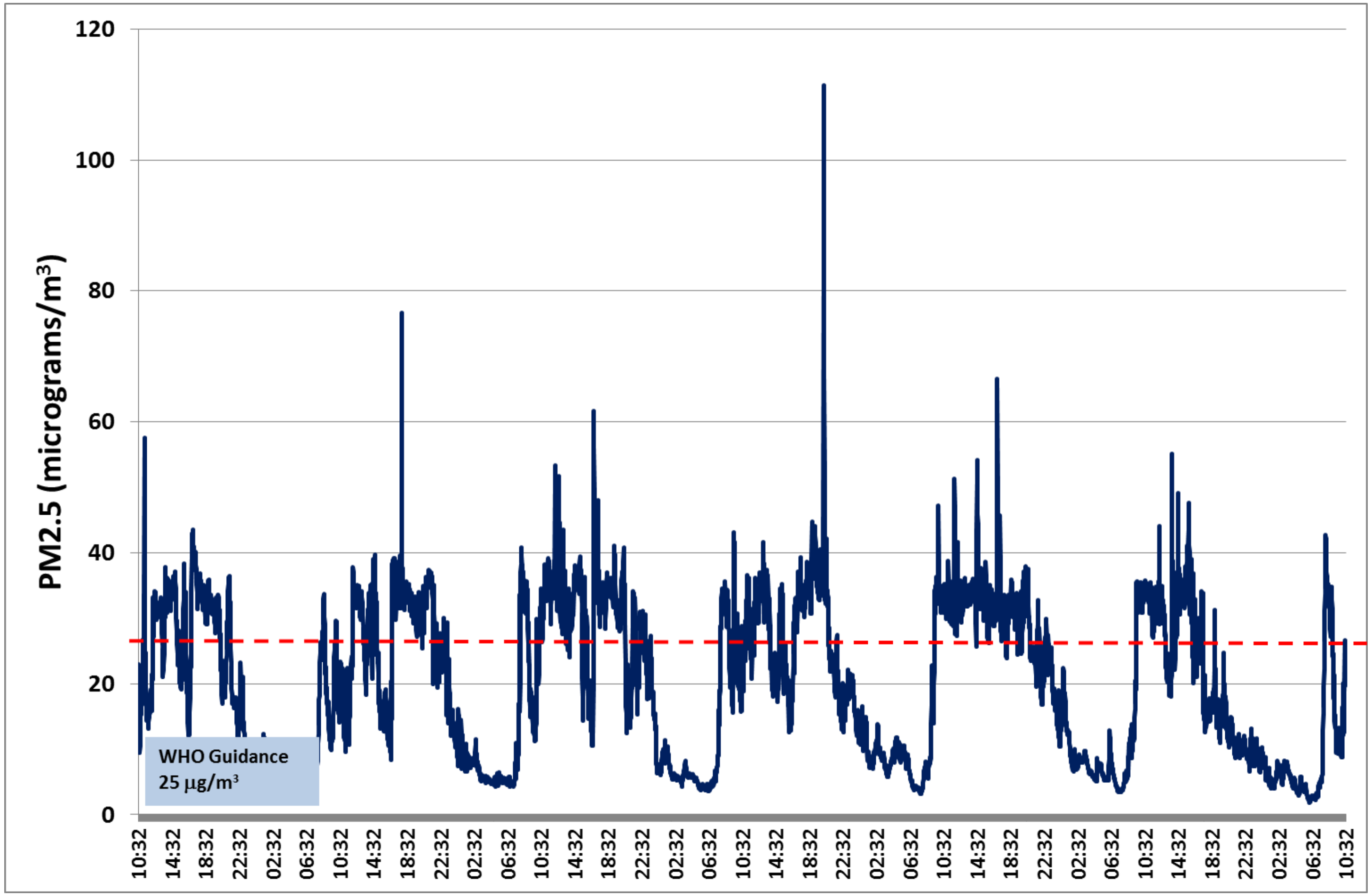

PM2.5 (micrograms/m³)
120
100
80
60
40
20
0
WHO Guidance
25 µg/m³
10:32
14:32
18:32
22:32
02:32
06:32
10:32
14:32
18:32
22:32
02:32
06:32
10:32
14:32
18:32
22:32
02:32
06:32
10:32
14:32
18:32
22:32
02:32
06:32
10:32
14:32
18:32
22:32
02:32
06:32
10:32
14:32
18:32
22:32
02:32
06:32
10:32

Prison 13

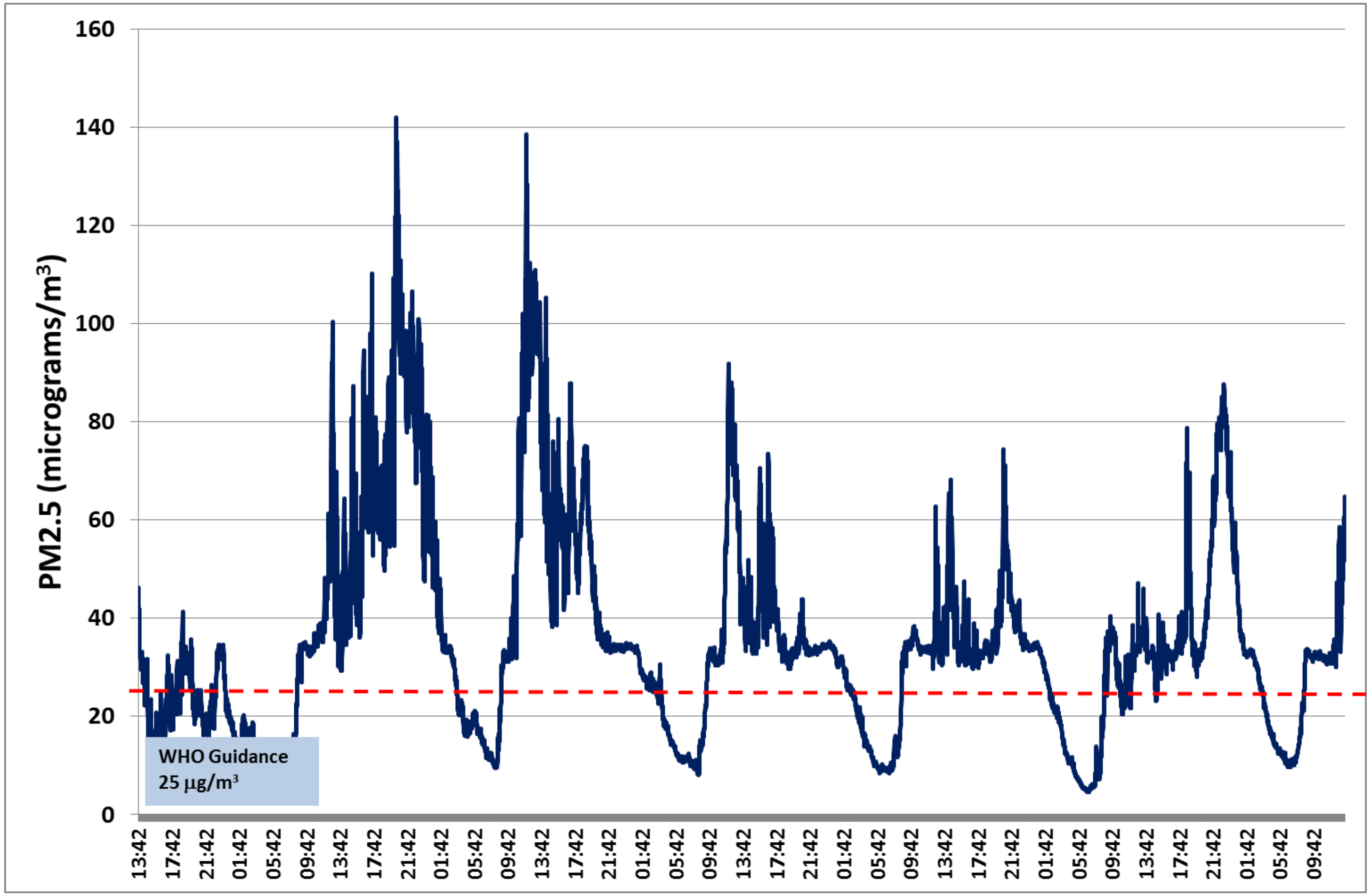

PM2.5 (micrograms/m3)
160
140
120
100
80
60
40
20
0
WHO Guidance
25 µg/m3
13:42
17:42
21:42
01:42
05:42
09:42
13:42
17:42
21:42
01:42
05:42
09:42
13:42
17:42
21:42
01:42
05:42
09:42
13:42
17:42
21:42
01:42
05:42
09:42
13:42
17:42
21:42
01:42
05:42
09:42
13:42
17:42
21:42
01:42
05:42
09:42

Prison 14

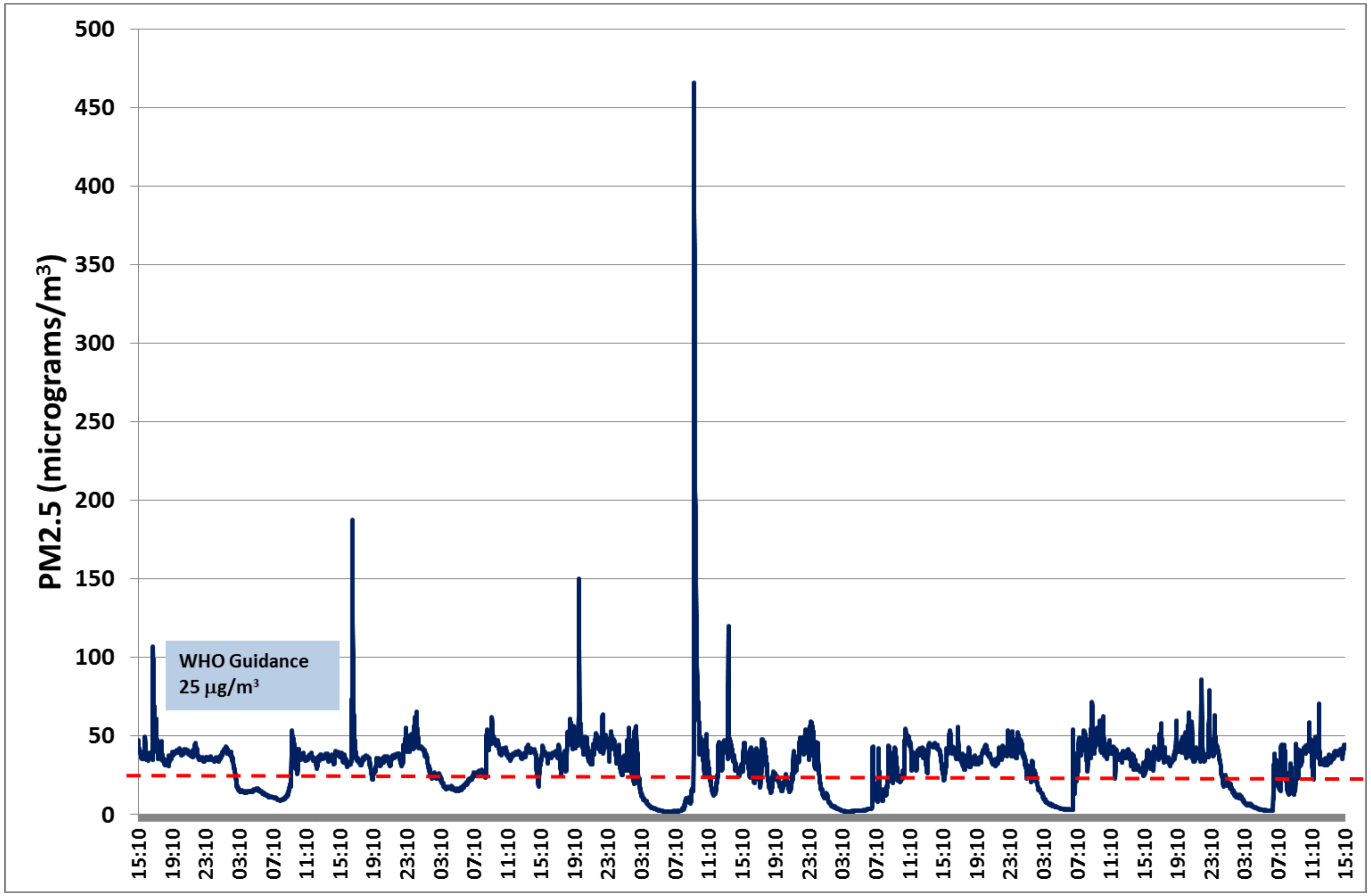

PM2.5 (micrograms/m³)
500
450
400
350
300
250
200
150
100
50
0
WHO Guidance
25 µg/m³
15:10
19:10
23:10
03:10
07:10
11:10
15:10
19:10
23:10
03:10
07:10
11:10
15:10
19:10
23:10
03:10
07:10
11:10
15:10
19:10
23:10
03:10
07:10
11:10
15:10
19:10
23:10
03:10
07:10
11:10
15:10
19:10
23:10
03:10
07:10
11:10
15:10

Prison 15

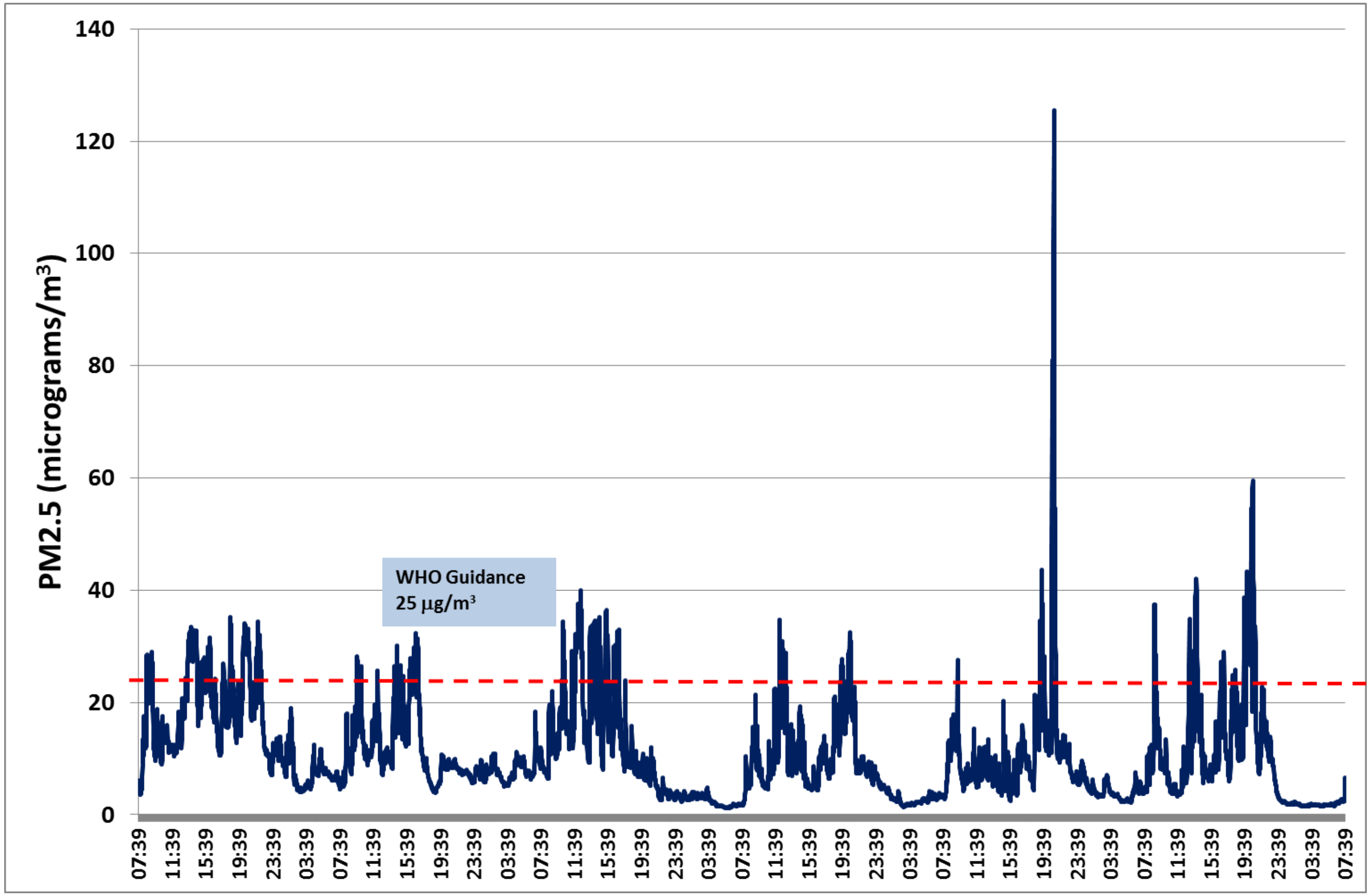

PM2.5 (micrograms/m3)
140
120
100
80
60
40
20
0
WHO Guidance
25 µg/m3
07:39
11:39
15:39
19:39
23:39
03:39
07:39
11:39
15:39
19:39
23:39
03:39
07:39
11:39
15:39
19:39
23:39
03:39
07:39
11:39
15:39
19:39
23:39
03:39
07:39
11:39
15:39
19:39
23:39
03:39
07:39
11:39
15:39
19:39
23:39
03:39
07:39

Mobile monitoring data graphical outputs

Prison 1

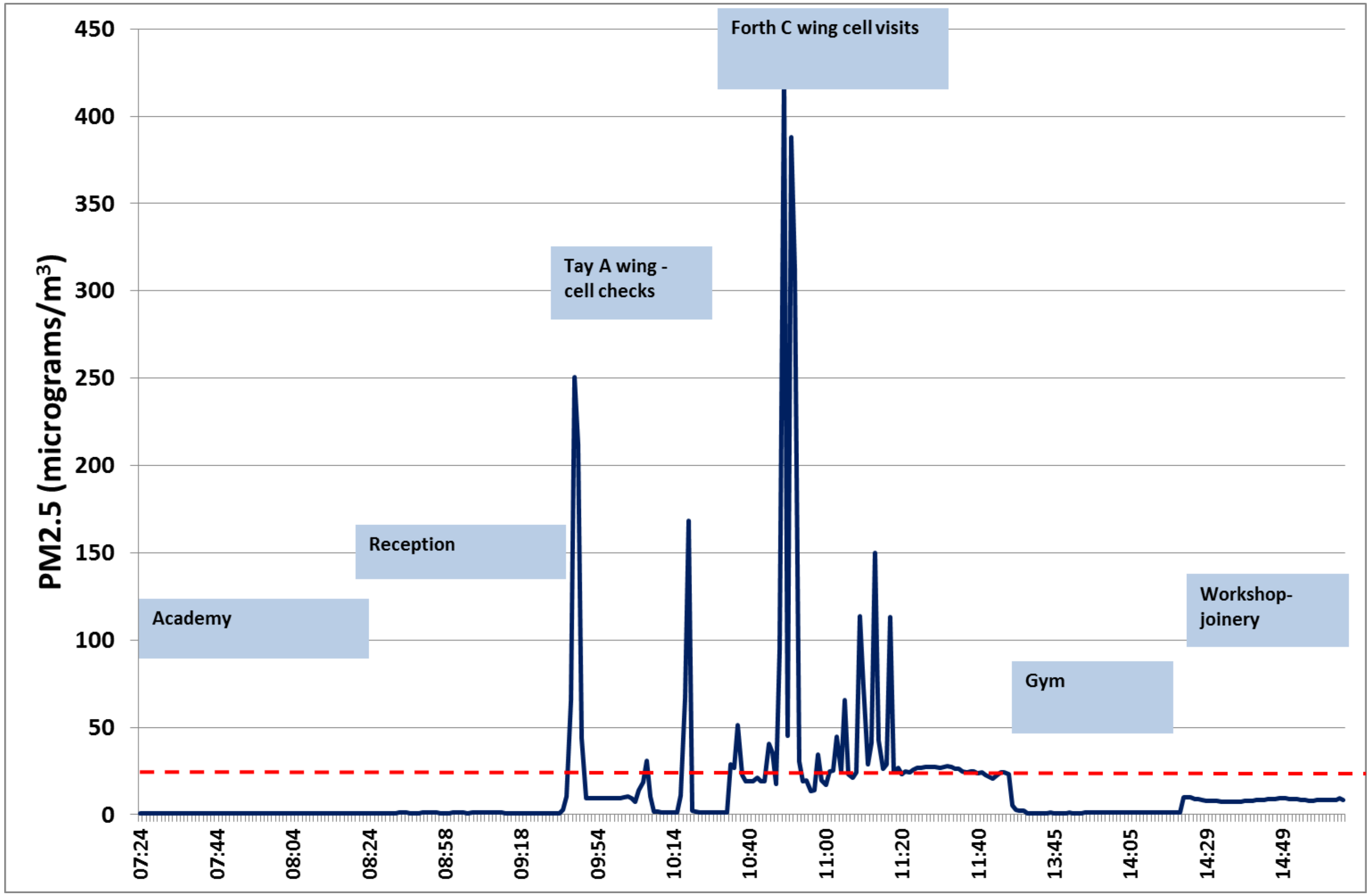

Prison 2

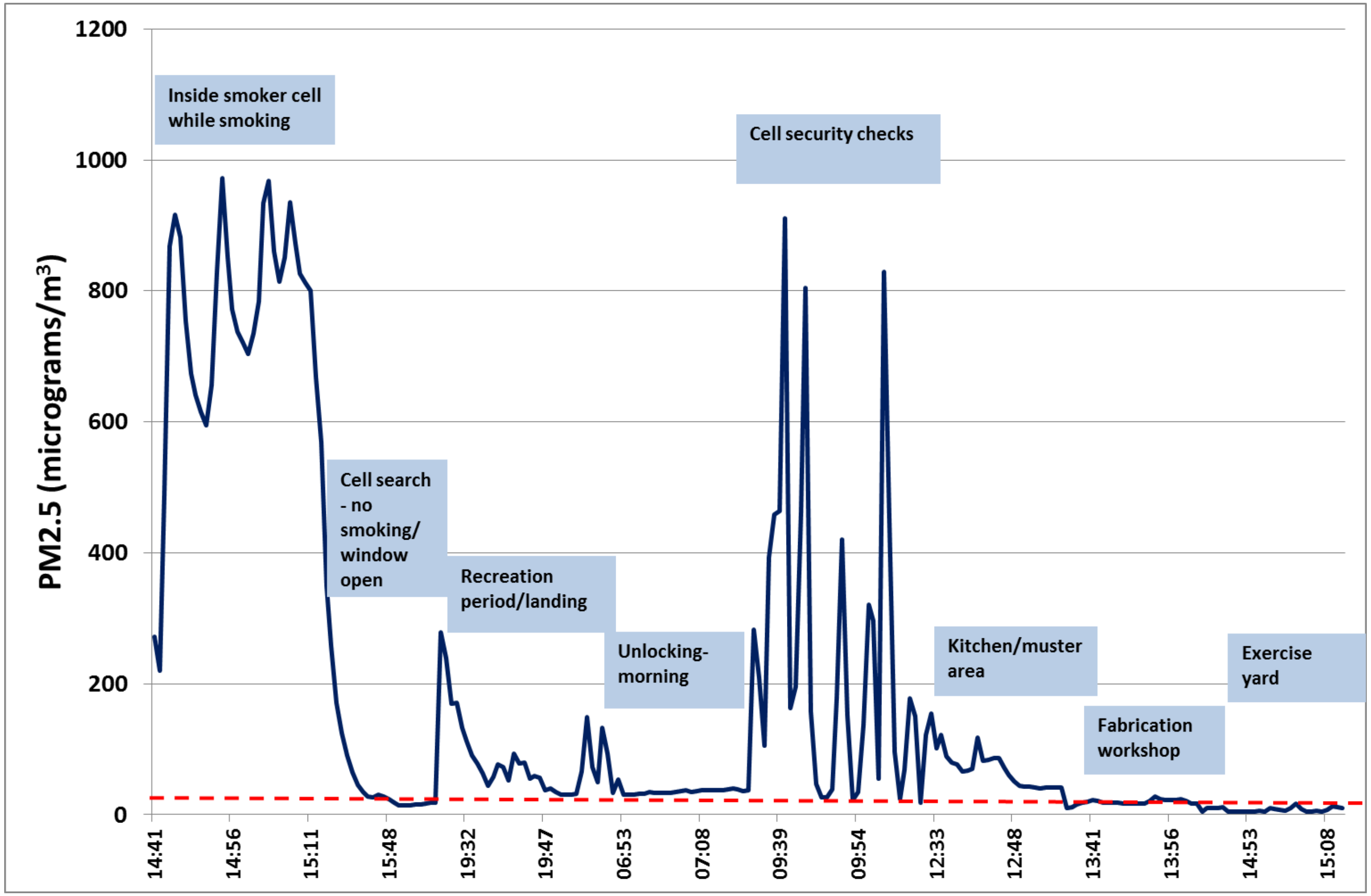

PM2.5 (micrograms/m³)
1200
1000
800
600
400
200
0
Inside smoker cell while smoking
Cell search - no smoking/ window open
Recreation period/landing
Unlocking- morning
Cell security checks
Kitchen/muster area
Fabrication workshop
Exercise yard
14:41
14:56
15:11
15:48
19:32
19:47
06:53
07:08
09:39
09:54
12:33
12:48
13:41
13:56
14:53
15:08

Prison 3

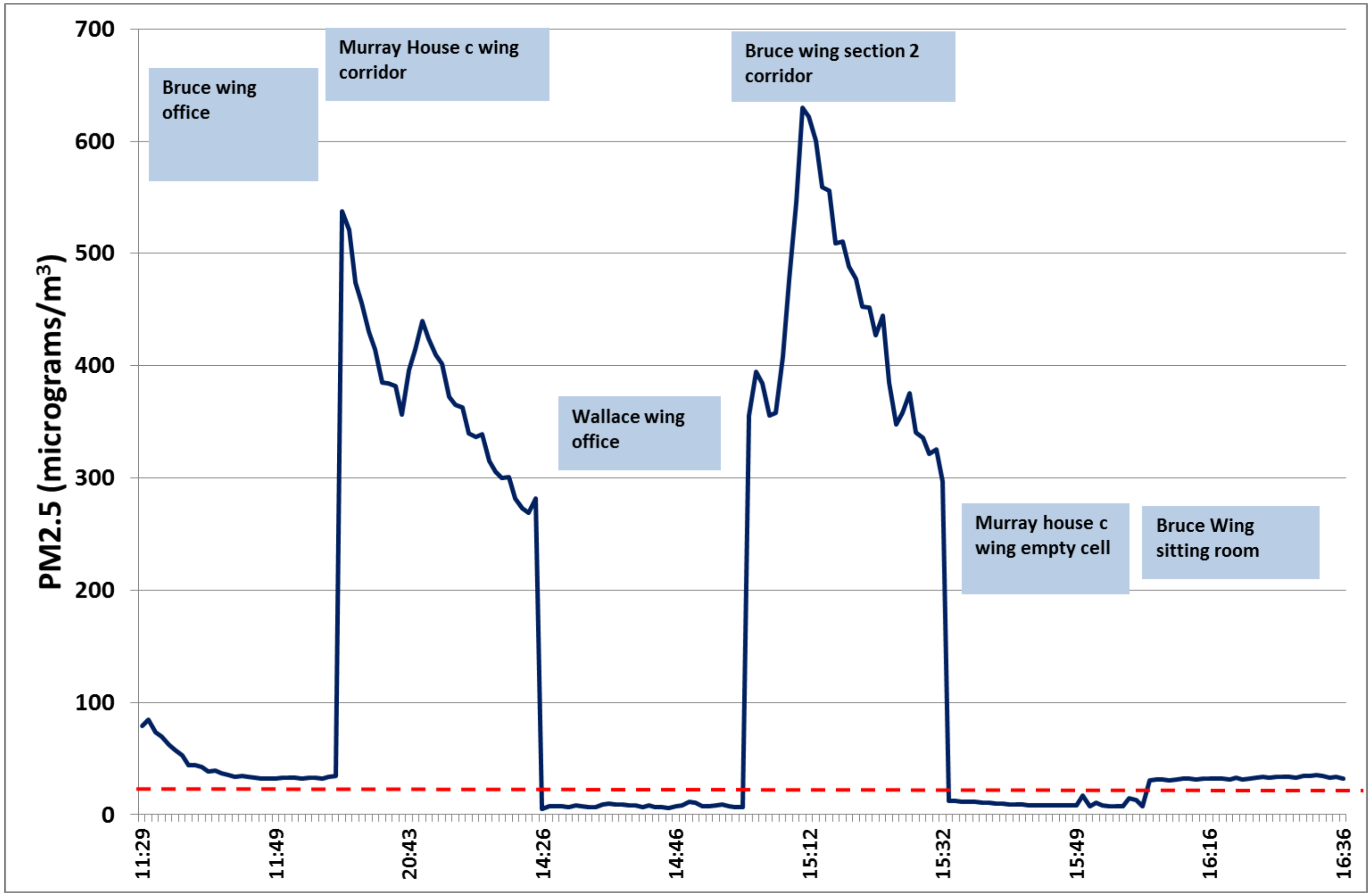

PM2.5 (micrograms/m3)
700
600
500
400
300
200
100
0
Bruce wing office
Murray House c wing corridor
Wallace wing office
Bruce wing section 2 corridor
Murray house c wing empty cell
Bruce Wing sitting room
11:29
11:49
20:43
14:26
14:46
15:12
15:32
15:49
16:16
16:36

Prison 4

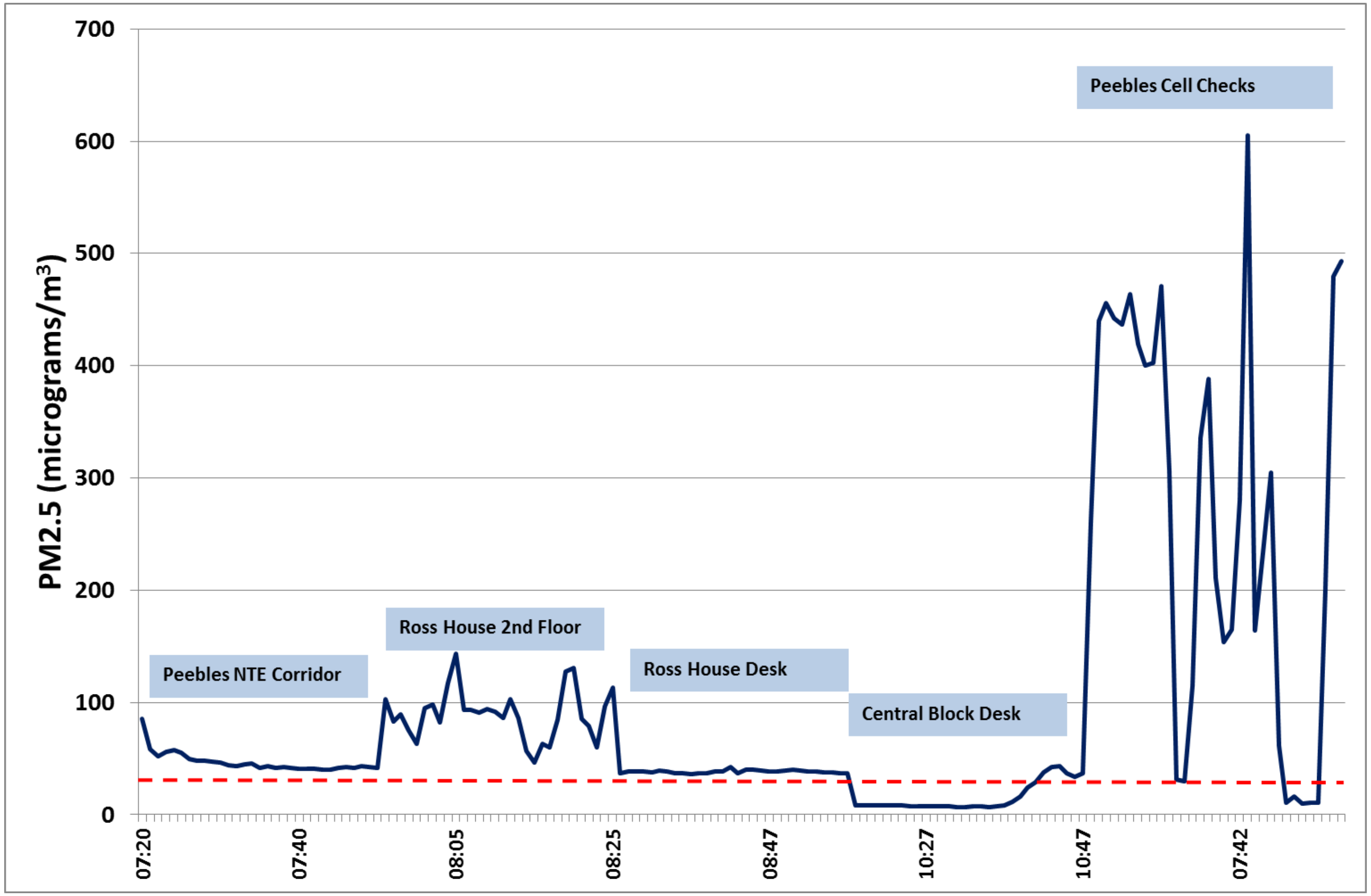

PM2.5 (micrograms/m3)
700
600
500
400
300
200
100
0
Peebles Cell Checks
Ross House 2nd Floor
Peebles NTE Corridor
Ross House Desk
Central Block Desk
07:20
07:40
08:05
08:25
08:47
10:27
10:47
07:42

Prison 5

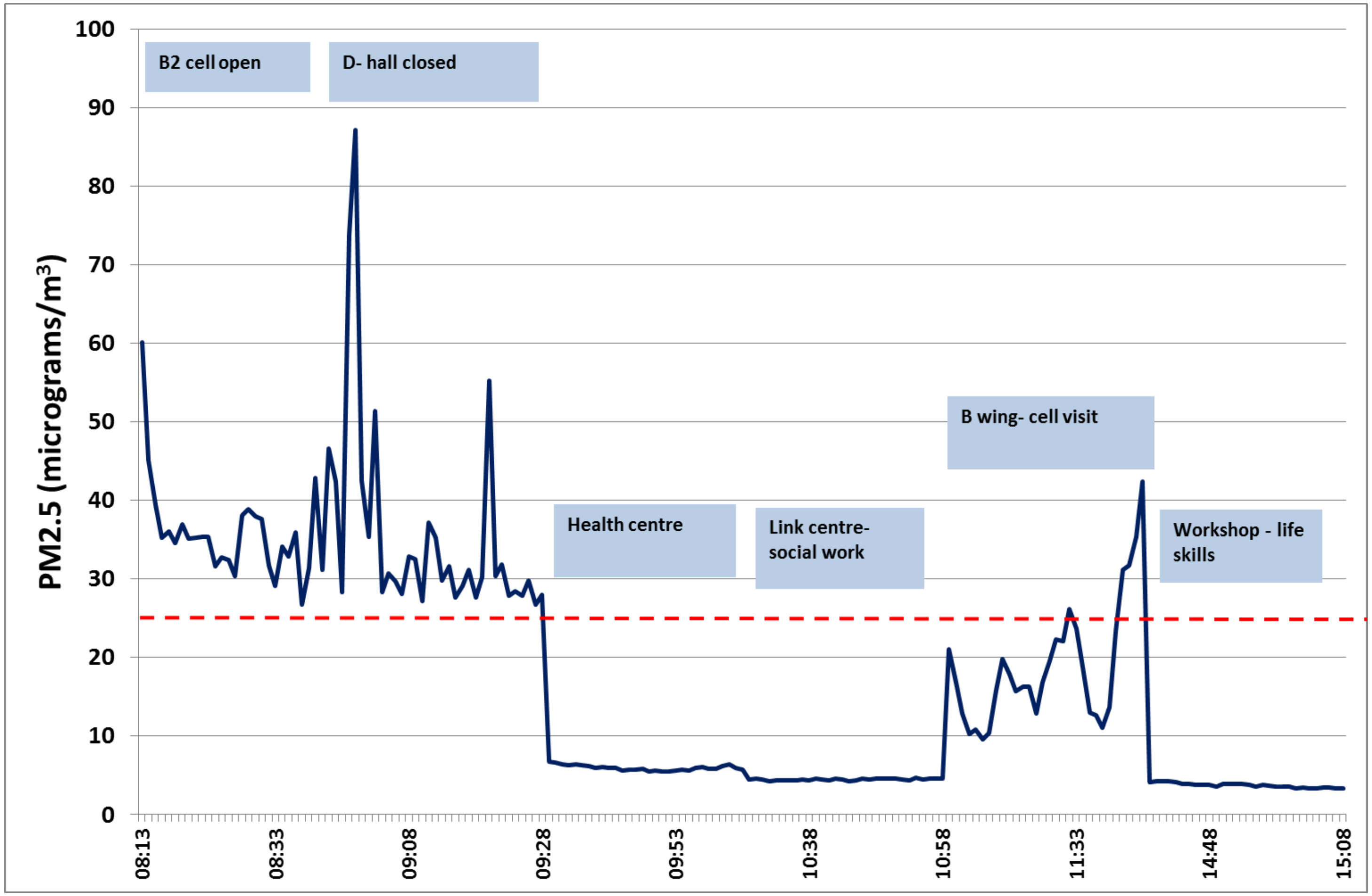

PM2.5 (micrograms/m³)
100
90
80
70
60
50
40
30
20
10
0
B2 cell open
D- hall closed
Health centre
Link centre- social work
B wing- cell visit
Workshop - life skills
08:13
08:33
09:08
09:28
09:53
10:38
10:58
11:33
14:48
15:08

Prison 6

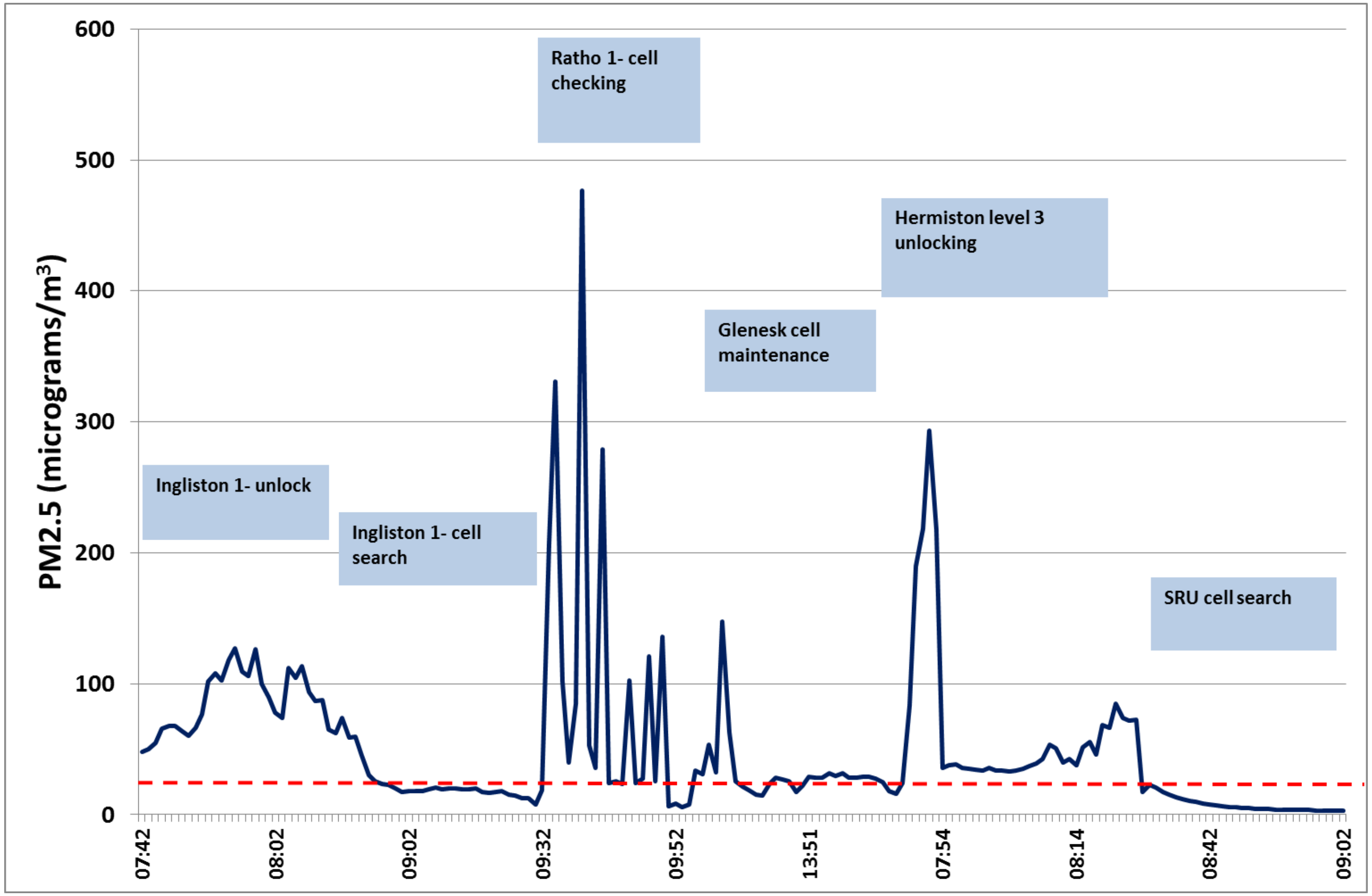

PM2.5 (micrograms/m³)
600
500
400
300
200
100
0
Ratho 1- cell checking
Hermiston level 3 unlocking
Glenesk cell maintenance
Ingliston 1- unlock
Ingliston 1- cell search
SRU cell search
07:42
08:02
09:02
09:32
09:52
13:51
07:54
08:14
08:42
09:02

Prison 7

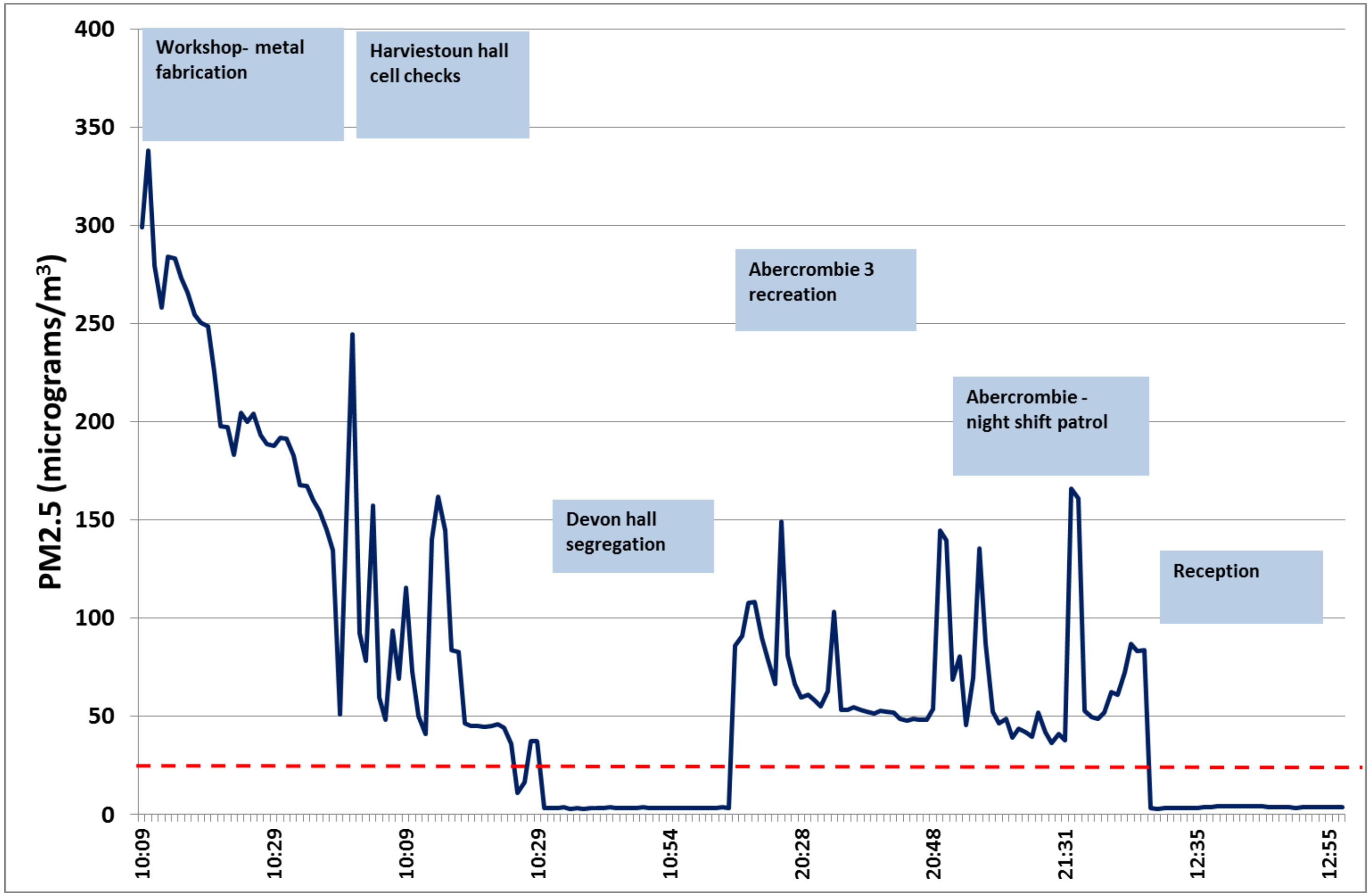

PM2.5 (micrograms/m3)
400
350
300
250
200
150
100
50
0
Workshop- metal fabrication
Harviestoun hall cell checks
Devon hall segregation
Abercrombie 3 recreation
Abercrombie - night shift patrol
Reception
10:09
10:29
10:09
10:29
10:54
20:28
20:48
21:31
12:35
12:55

Prison 8

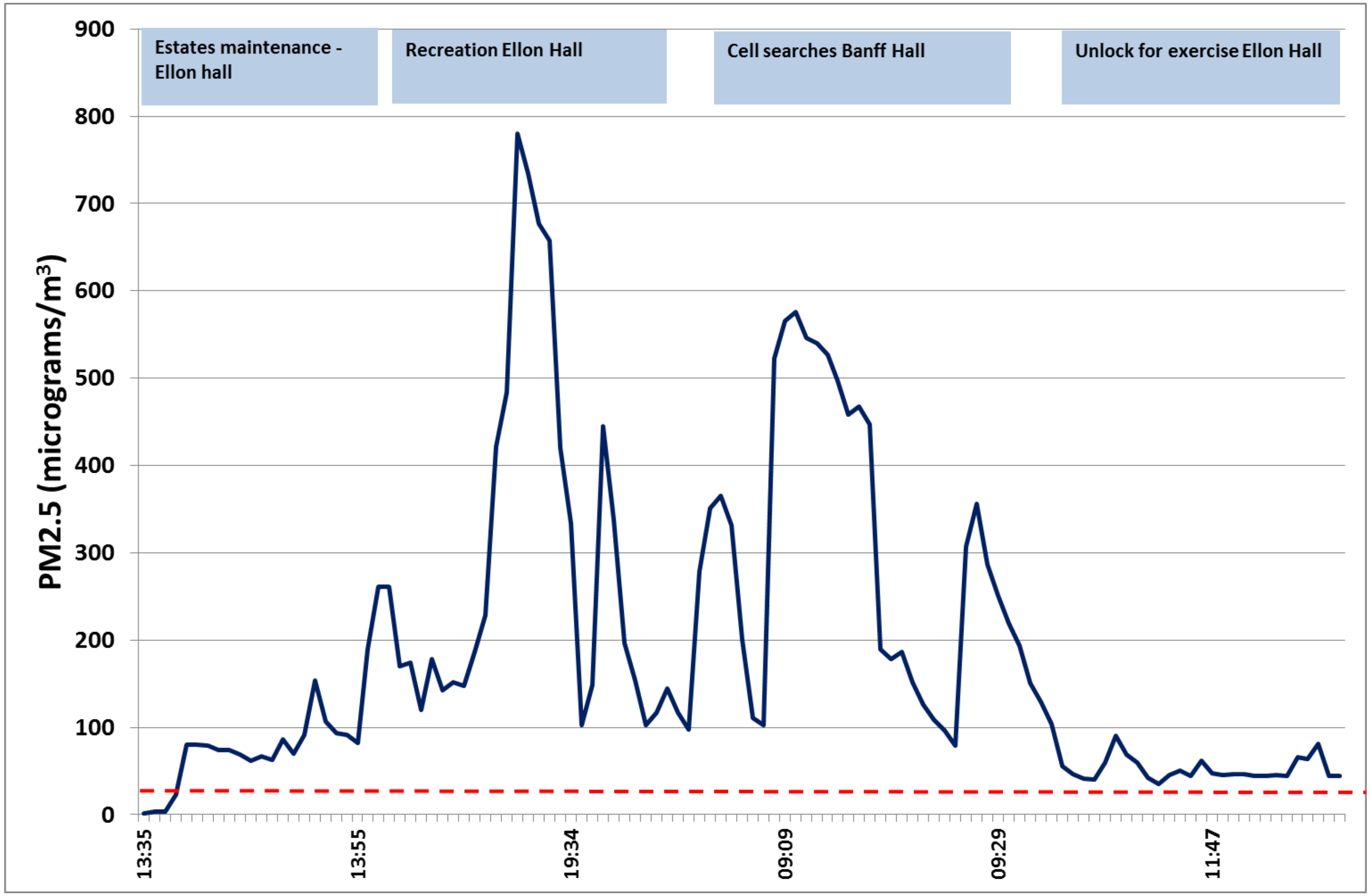

PM2.5 (micrograms/m3)
900
800
700
600
500
400
300
200
100
0
Estates maintenance - Ellon hall
Recreation Ellon Hall
Cell searches Banff Hall
Unlock for exercise Ellon Hall
13:35
13:55
19:34
09:09
09:29
11:47

Prison 9

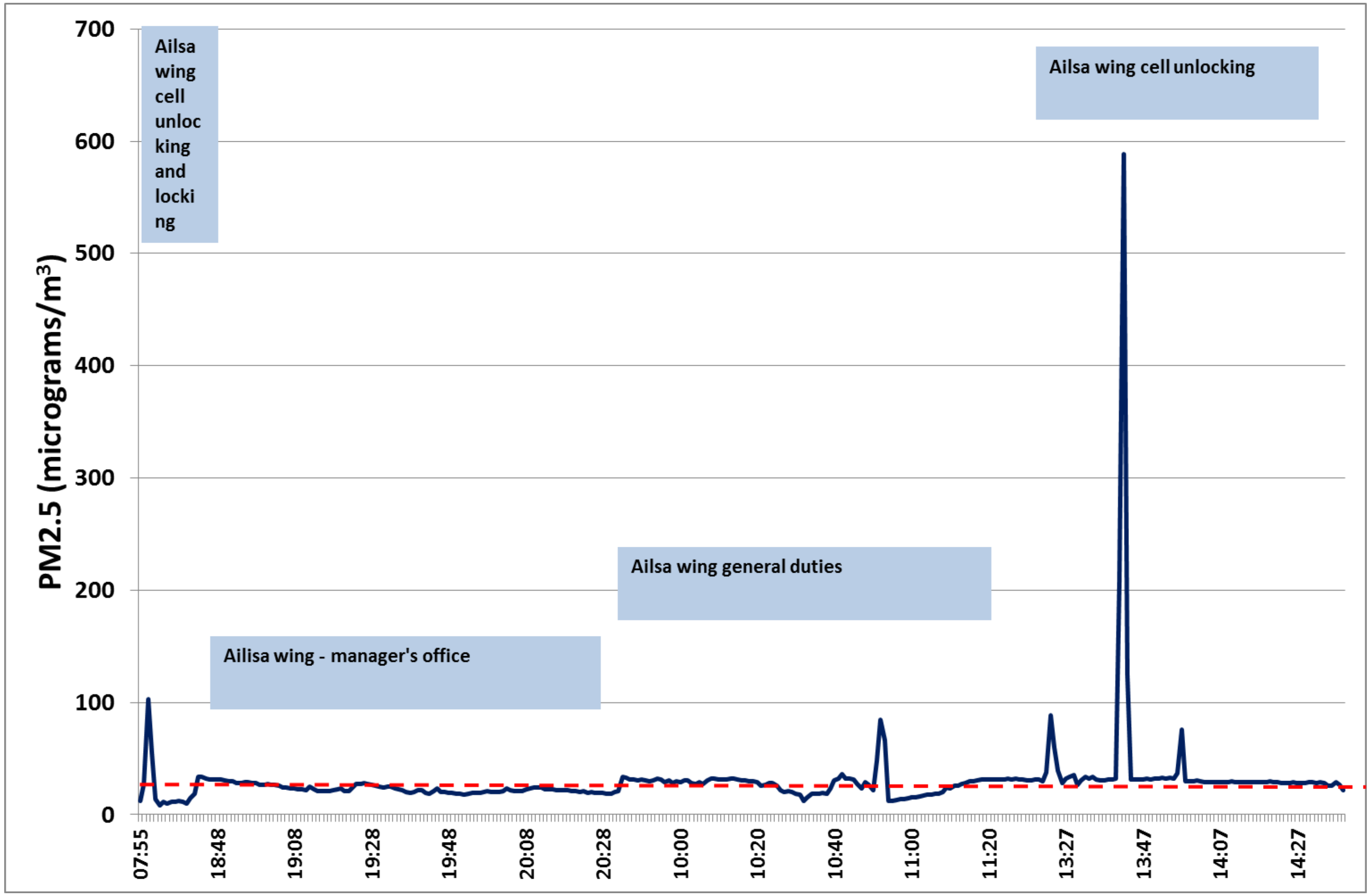

PM2.5 (micrograms/m³)
700
600
500
400
300
200
100
0
Ailsa wing cell unlocking and locking
Ailsa wing cell unlocking
Ailsa wing general duties
Ailisa wing - manager's office
07:55
18:48
19:08
19:28
19:48
20:08
20:28
10:00
10:20
10:40
11:00
11:20
13:27
13:47
14:07
14:27

Prison 10

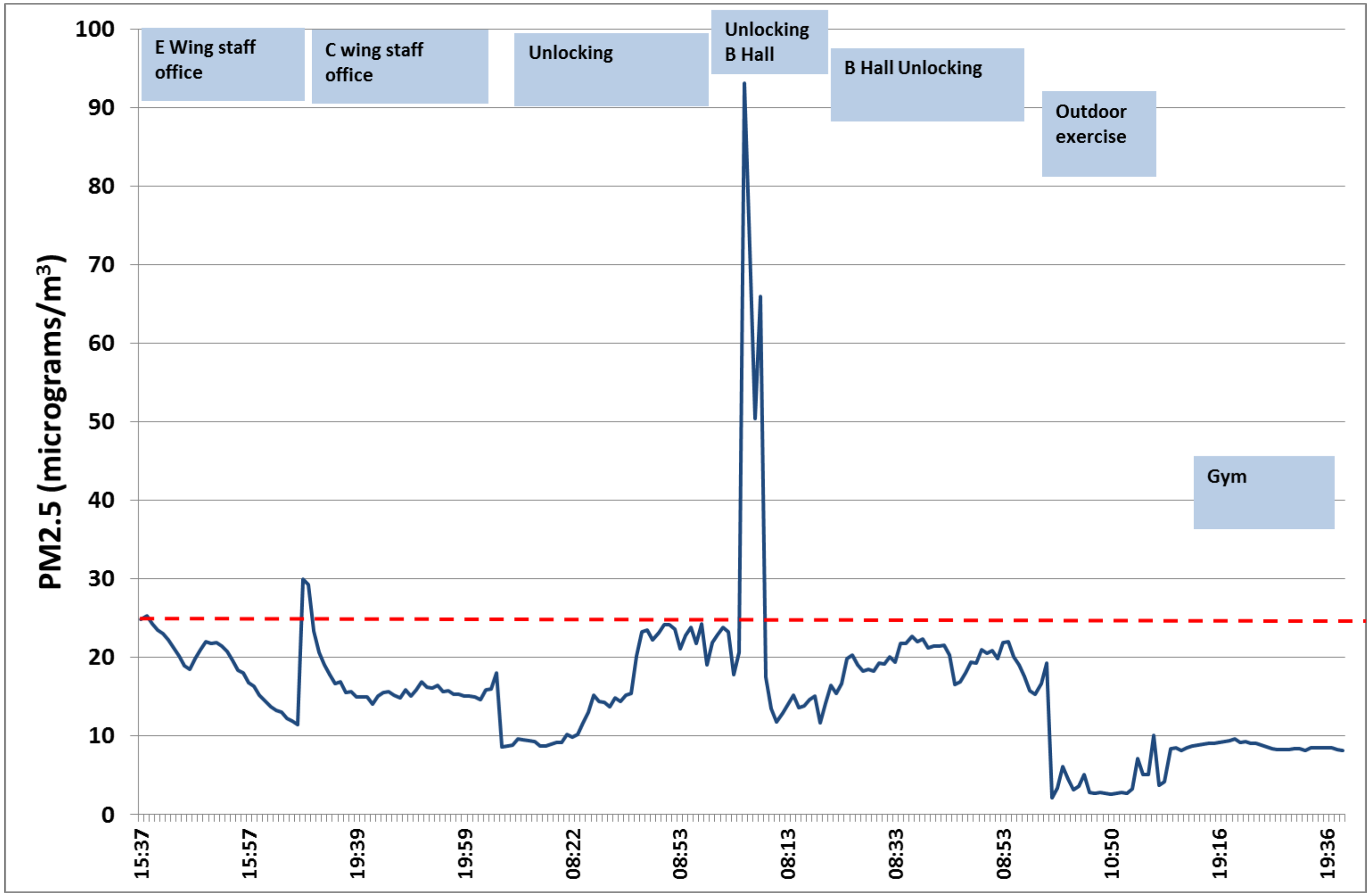

E Wing staff office
C wing staff office
Unlocking
Unlocking B Hall
B Hall Unlocking
Outdoor exercise
Gym
PM2.5 (micrograms/m³)
100
90
80
70
60
50
40
30
20
10
0
15:37
15:57
19:39
19:59
08:22
08:53
08:13
08:33
08:53
10:50
19:16
19:36

Prison 11

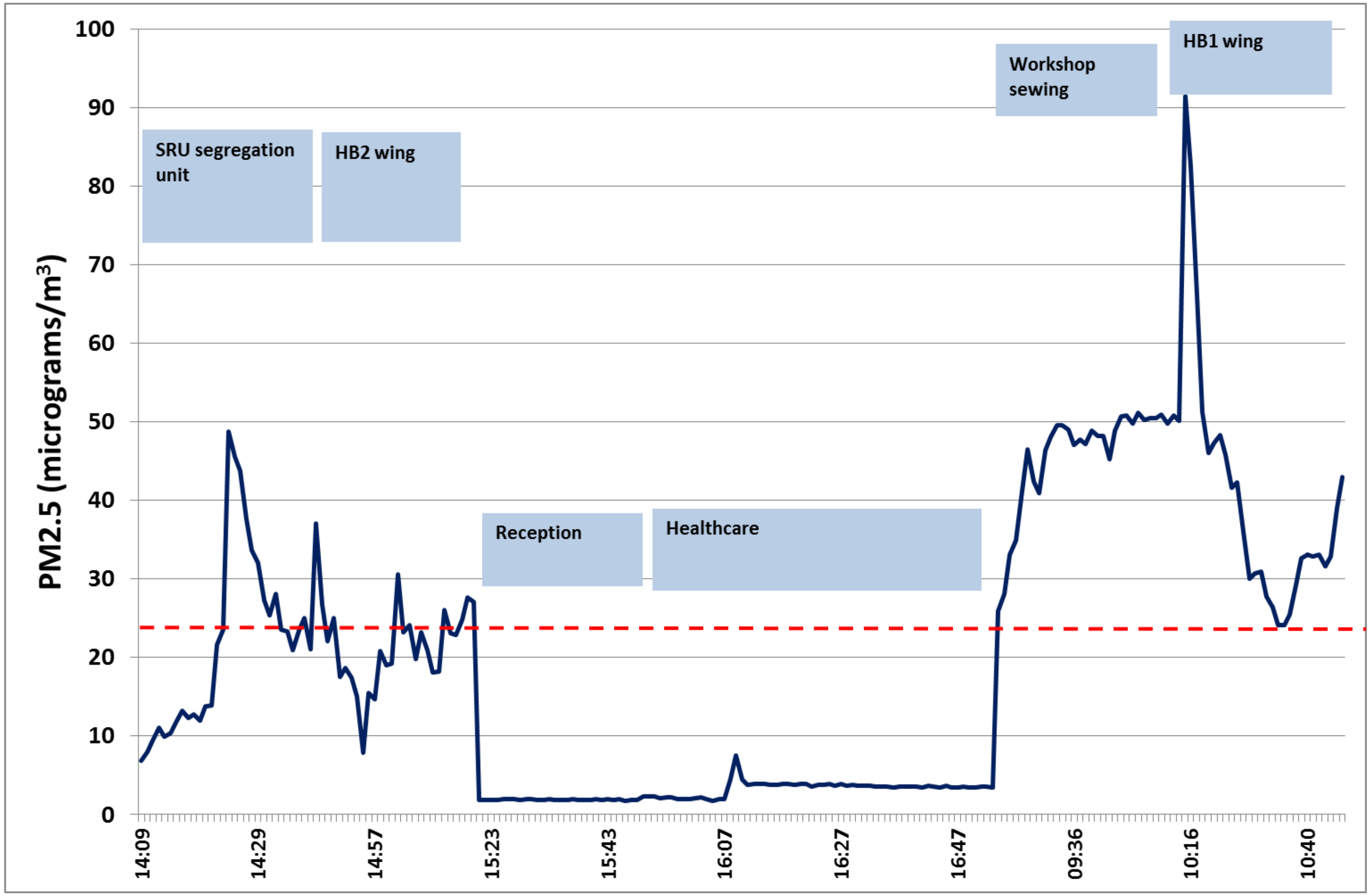
PM2.5 (micrograms/m³)
100
90
80
70
60
50
40
30
20
10
0
SRU segregation unit
HB2 wing
Reception
Healthcare
Workshop sewing
HB1 wing
14:09
14:29
14:57
15:23
15:43
16:07
16:27
16:47
09:36
10:16
10:40

Prison 12

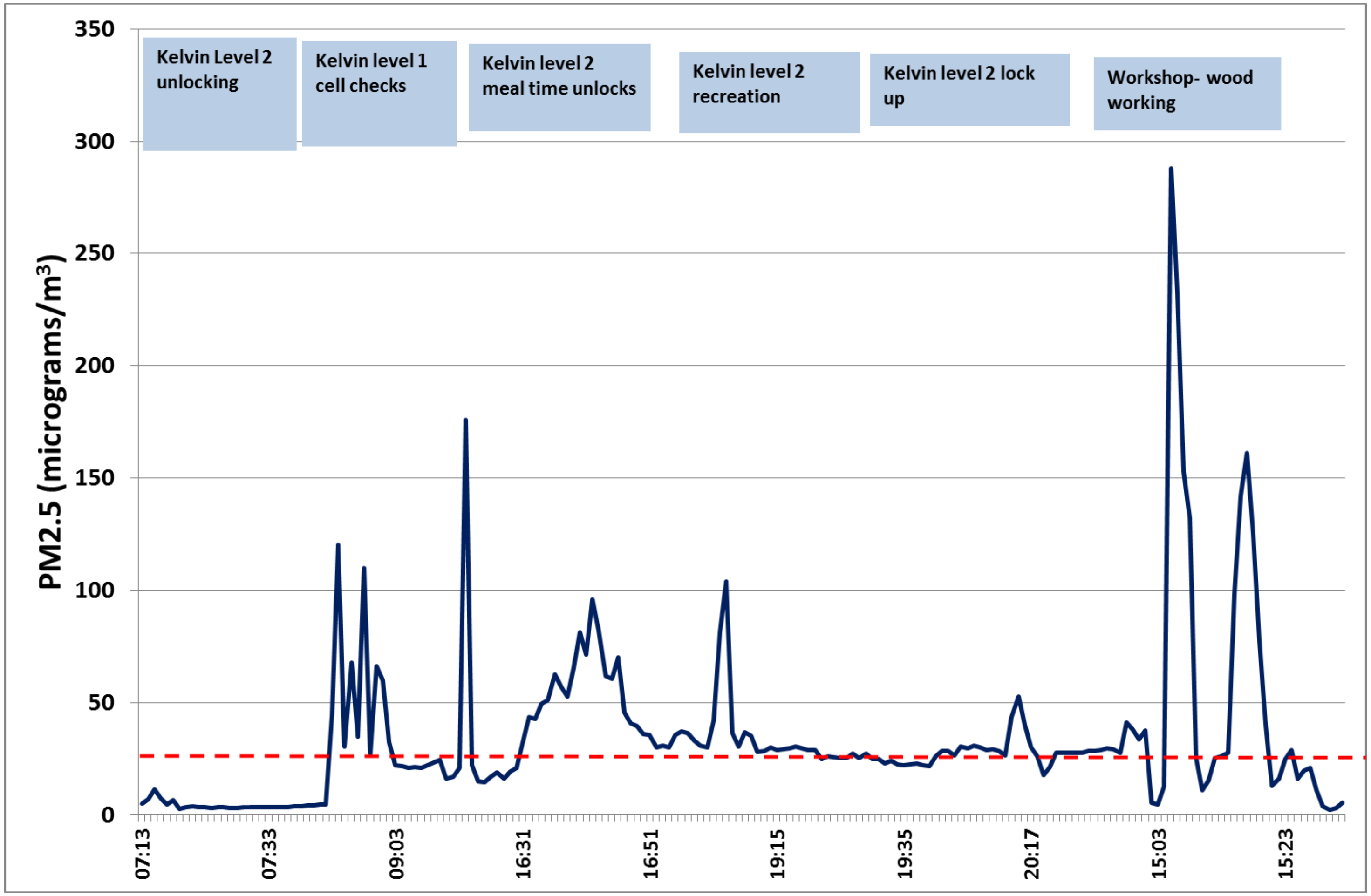

Kelvin Level 2 unlocking
Kelvin level 1 cell checks
Kelvin level 2 meal time unlocks
Kelvin level 2 recreation
Kelvin level 2 lock up
Workshop- wood working
PM2.5 (micrograms/m3)
350
300
250
200
150
100
50
0
07:13
07:33
09:03
16:31
16:51
19:15
19:35
20:17
15:03
15:23

Prison 13

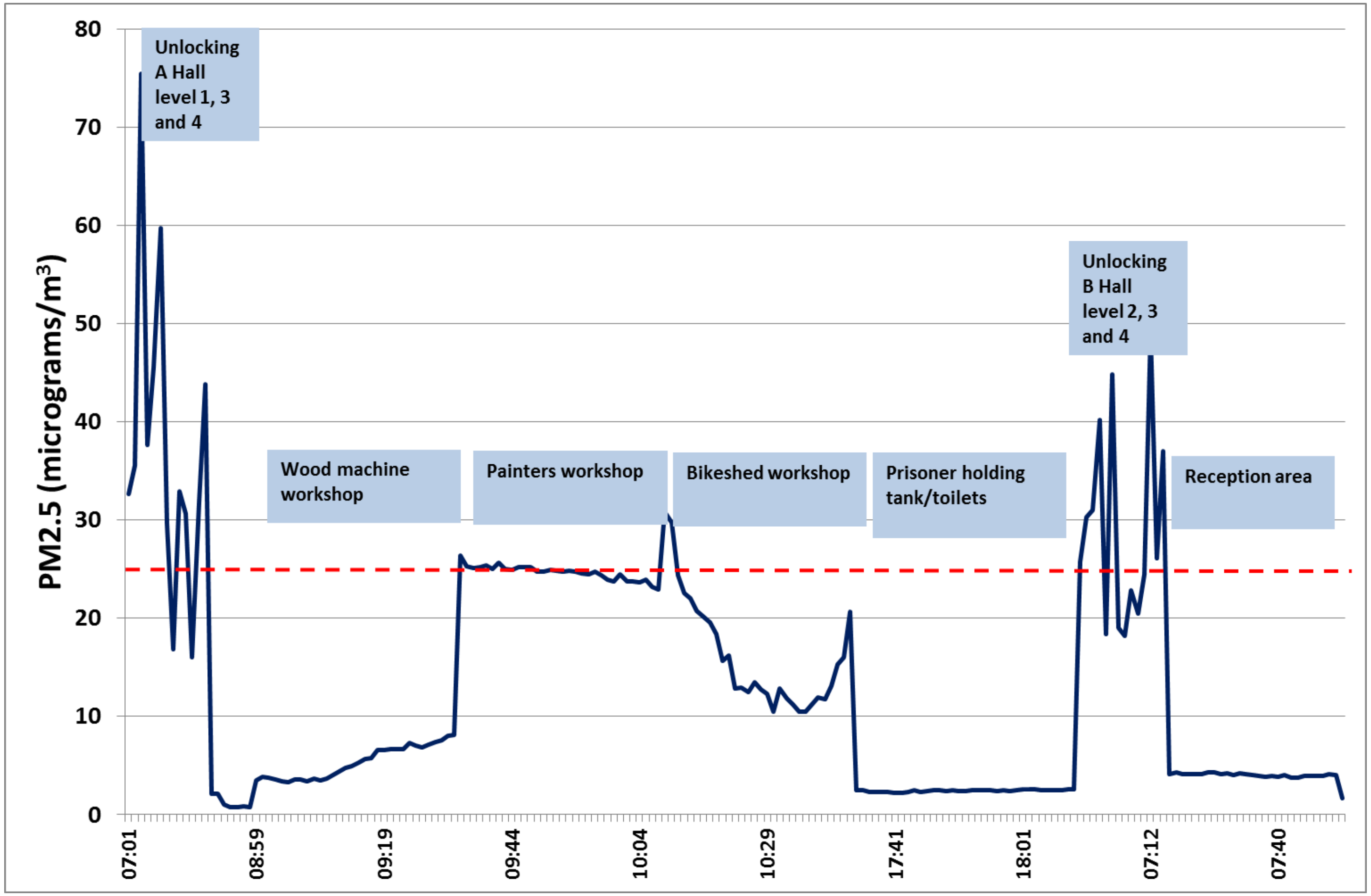

80
70
60
50
40
30
20
10
0
PM2.5 (micrograms/m3)
Unlocking A Hall level 1, 3 and 4
Wood machine workshop
Painters workshop
Bikeshed workshop
Prisoner holding tank/toilets
Unlocking B Hall level 2, 3 and 4
Reception area
07:01
08:59
09:19
09:44
10:04
10:29
17:41
18:01
07:12
07:40

Prison 14

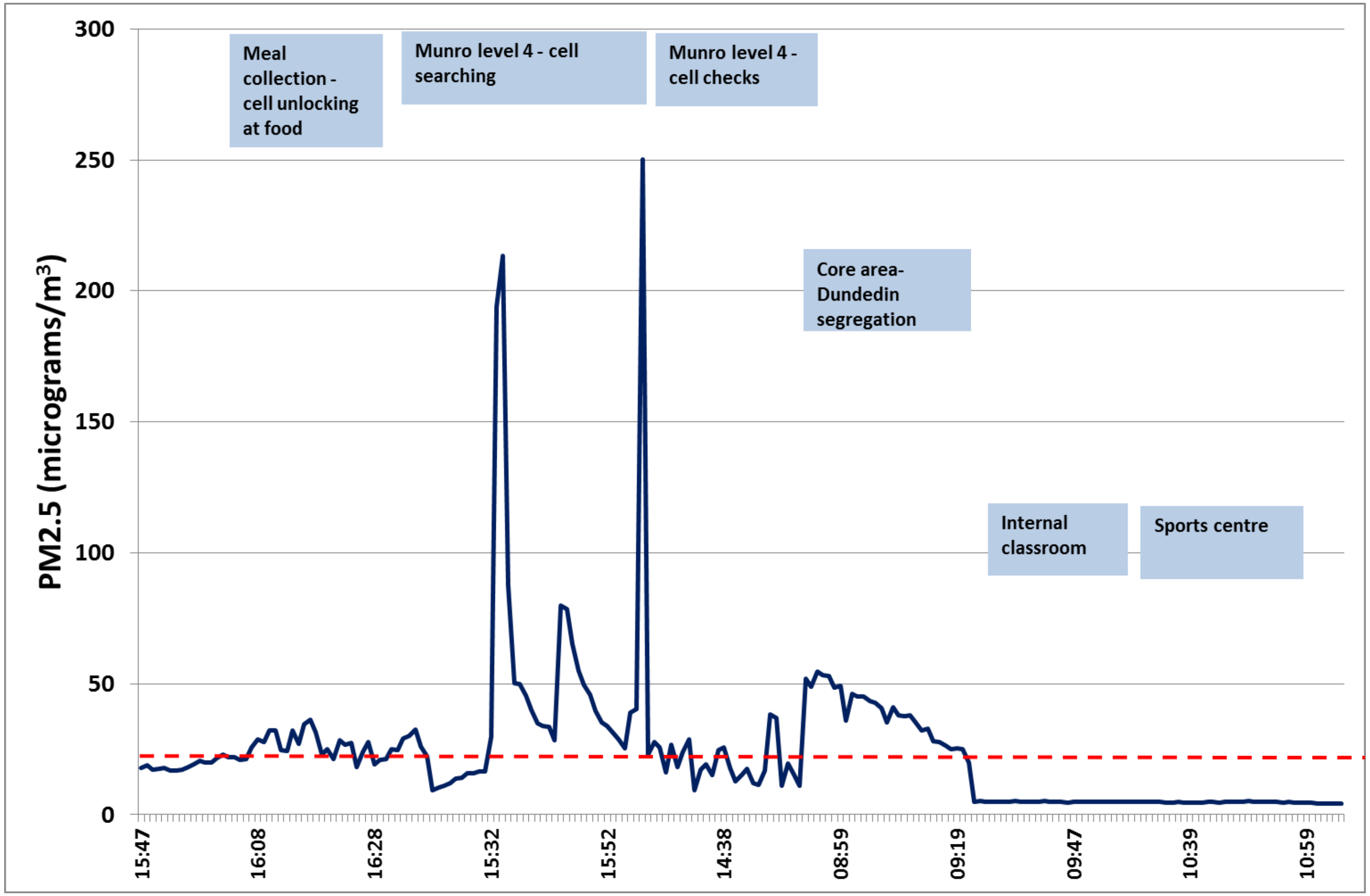

PM2.5 (micrograms/m3)
300
250
200
150
100
50
0
Meal collection - cell unlocking at food
Munro level 4 - cell searching
Munro level 4 - cell checks
Core area- Dundedin segregation
Internal classroom
Sports centre
15:47
16:08
16:28
15:32
15:52
14:38
08:59
09:19
09:47
10:39
10:59

Prison 15

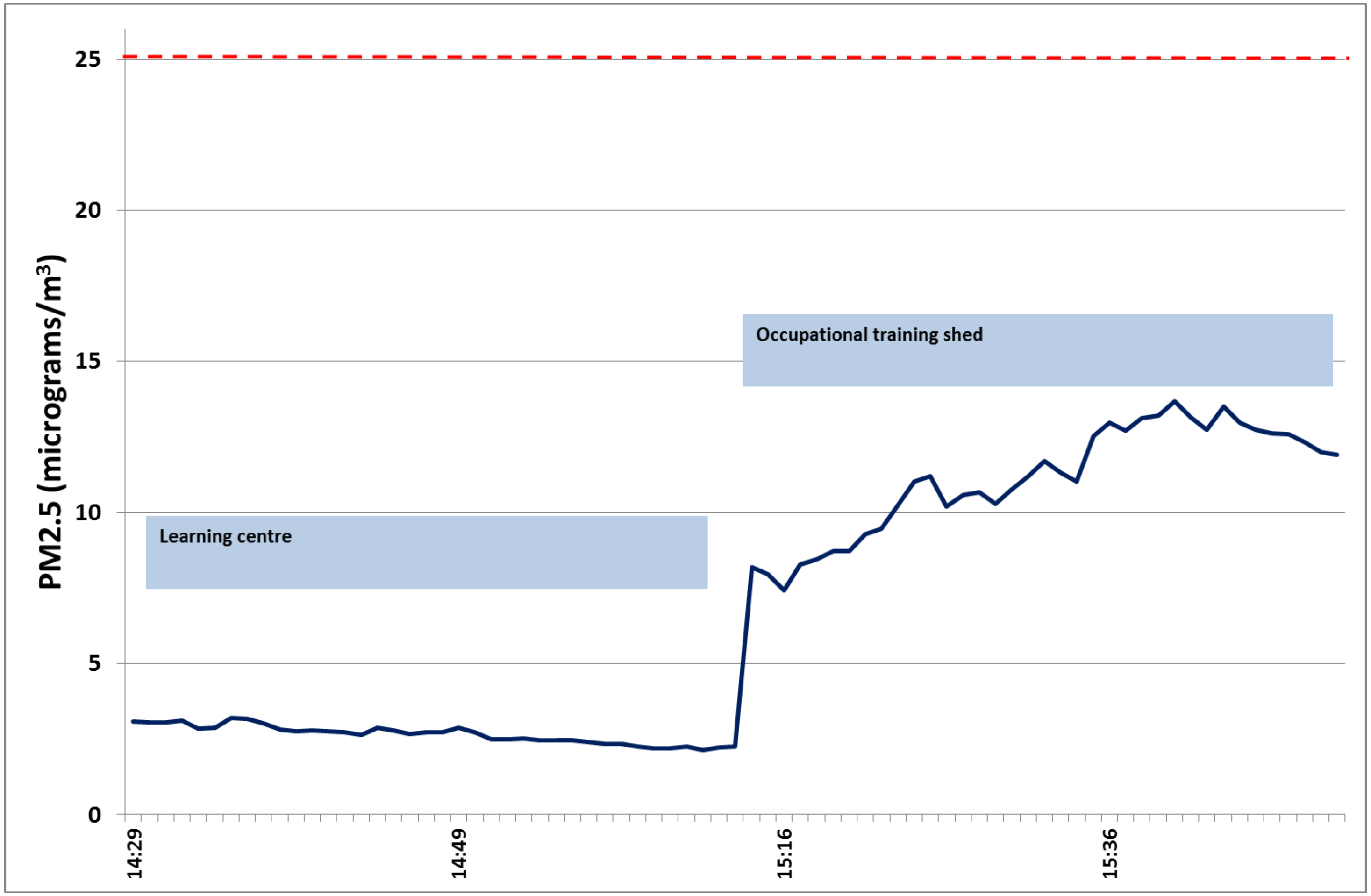

PM2.5 (micrograms/m3)
25
20
15
10
5
0
Learning centre
Occupational training shed
14:29
14:49
15:16
15:36
